# Expanding the *RB1* variant landscape of heritable retinoblastoma: unlocking precision oncology potential in Southern Africa

**DOI:** 10.1186/s12885-026-15789-7

**Published:** 2026-02-28

**Authors:** Indiana van Rensburg, Hamzah Mustak, Gameda Benefeld, Lucy Cunnama, Helga Abrahamse-Pillay, Raj Ramesar, Lisa Roberts

**Affiliations:** 1 UCT/MRC Genomic and Precision Medicine Research Unit, Division of Human Genetics, Department of Pathology, University of Cape Town, Cape Town, South Africa; 2 Division of Ophthalmology, Groote Schuur Hospital and University of Cape Town, Cape Town, South Africa; 3 Health Economics Unit and Division, School of Public Health, Faculty of Health Sciences, University of Cape Town, Cape Town, South Africa; 4 Division of Ophthalmology, Tygerberg Hospital and Stellenbosch University, Cape Town, South Africa

**Keywords:** Retinoblastoma, Cancer genetics, Next-generation sequencing, Copy number variant analysis, Molecular diagnosis, Health economics

## Abstract

**Background:**

Retinoblastoma (Rb) is the most common childhood intraocular cancer, with heritable forms caused by pathogenic germline variants in the *RB1* (or retinoblastoma 1) gene, which necessitate intensive and costly lifelong surveillance. Genetic testing enables precise risk prediction and individualized clinical management, yet data on the *RB1* variant spectrum in African populations remains scarce. This study investigated the molecular landscape of clinically presumed heritable Rb in a Southern African cohort and evaluated the potential healthcare and economic benefits of implementing genetic testing.

**Methods:**

Fifty-eight patients with clinically presumed heritable Rb were recruited from two tertiary hospitals in South Africa. Comprehensive *RB1* gene analysis was performed using next-generation sequencing (NGS) to detect single nucleotide variants (SNVs), small insertions and deletions (indels), and copy number variants (CNVs), and was supplemented by methylation-specific multiplex ligation-dependent probe amplification (MS-MLPA). A cost comparison model was developed to evaluate conventional clinical screening versus a genetically guided approach.

**Results:**

*RB1* pathogenic and/or likely pathogenic variants were identified in 84.5% (49/58) of patients, including eleven novel variants. The variant spectrum included frameshift, nonsense, splicing, intronic, and missense changes, with a notable concentration in the pocket domain region of the Rb associated protein (pRb). CNVs were detected in 10.3% of probands. Our cost analysis demonstrated significant savings associated with genetic testing, reducing surveillance-related expenses by up to US$228,989 across extended family generations in the private sector and US$166,489 in the public sector. Implementing genetic testing would also minimize unnecessary examinations and promote more efficient allocation of healthcare resources.

**Conclusions:**

Comprehensive *RB1* genetic testing in this Southern African cohort revealed a diverse variant landscape, including novel pathogenic changes, and demonstrated the clinical and economic value of integrating genetic testing into Rb management. These findings contribute to the global understanding of genetic variation within the *RB1* gene and support tailored care strategies that can reduce the surveillance burden, optimize healthcare resources, and improve outcomes for affected families.

**Supplementary Information:**

The online version contains supplementary material available at 10.1186/s12885-026-15789-7.

## Background

Retinoblastoma (Rb), a malignant tumor of the eye, is the most prevalent childhood intraocular cancer and one of six paediatric cancers prioritized by the World Health Organization (WHO) for global action [1, 2]. Worldwide, Rb affects approximately 1 in 15,000 to 20,000 live births [3]. In South Africa (SA) the age-standardized incidence rate is around 1 in 21,641 live births; an estimate likely impacted by incomplete capture of all cases within the healthcare system, including variation in access to specialized care [4]. Although Rb is among the most successfully treated pediatric cancers in developed countries, the global burden remains substantial, with approximately 89% of affected children living in low- and middle-income countries (LMICs) [4, 5]. While mortality from Rb is rare in developed countries, estimated three-year survival rates in low-income countries remain just above 50% [6]. This disparity is largely driven by delayed diagnosis, which often leads to advanced disease at presentation, reducing the likelihood of eye preservation and significantly increasing the risk of metastasis [7]. These inequities highlight the urgent need for targeted research and intervention in SA and other LMICs to address healthcare gaps and improve patient outcomes.

The study of Rb has been instrumental in advancing our understanding of cancer genetics. In 1971, Alfred Knudson proposed the “two-hit” hypothesis, suggesting that Rb arises from the biallelic inactivation of a tumor suppressor gene [8]. This work laid the foundation for the discovery and cloning of the *RB1 *(or retinoblastoma 1) gene in 1986, establishing it as the first tumor suppressor gene identified and fundamentally shifting the paradigm of hereditary cancer [9]. Located on the long arm of chromosome 13 (13q14.2), the *RB1 *gene consists of 27 exons spanning approximately 178 kb of genomic DNA, and encodes the Rb-associated protein (pRB), a key regulator of the cell cycle [10]. To date, 1799 unique DNA variants have been reported in the *RB1 *gene [11], ranging from single-base substitutions to large deletions [12]. Most causal *RB1 *variants are null variants that result in the loss of functional pRB protein [12, 13]. These null variants, which include frameshift, splice-site, and nonsense variants, as well as large deletions, account for approximately 90% of causal *RB1 *variants. Less common contributing variants include promoter, missense, and in-frame variants [12, 13].

Approximately 40% of Rb cases are heritable, with the pathogenic variant transmitted as a highly penetrant autosomal dominant trait, while the remaining 60% are non-heritable i.e. sporadic. In heritable Rb, the first variant occurs in the germline, affecting all bodily cells, while the second arises somatically in susceptible retinal cells [13]. This germline variant is either inherited from an affected parent (hereditary familial Rb) or arises spontaneously during gamete formation or early embryonic development (hereditary de novo Rb). Since only a single somatic variant is required to initiate Rb tumor development in heritable cases, individuals with this form of the disease often develop bilateral disease and multifocal tumors [14]. The predisposition to Rb typically follows an autosomal dominant inheritance pattern with high penetrance (90%) [15]. However, rare familial cases have also been described, characterized by low penetrance (where carriers remain unaffected) and variable expressivity (where carriers develop unilateral disease and unifocal tumors) [15].

It is recommended that individuals with Rb undergo an intensive and costly eye surveillance protocol, which includes frequent examinations under anesthetic (EUA) during early childhood. In accordance with South African guidelines, screening generally begins one week after birth, followed by monthly assessments for the first three months, every two months for the next six months, every three months for two years, and then every six months thereafter [16]. This rigorous schedule is essential for early tumor detection but places a significant financial and emotional burden on families, strains the healthcare system, and exposes children to repeated anesthesia with its associated risks [16]. In practice, all clinical screening in SA currently occurs in the absence of genetic testing, as diagnostic *RB1* testing is not yet available locally. Adherence to the recommended surveillance schedule is highly variable, with families often facing substantial barriers including long travel distances to tertiary centers, financial constraints, and the emotional and logistical burden of repeated EUAs. These challenges can contribute to delayed presentation and incomplete follow-up, highlighting the urgent need for accessible genetic testing. Additionally, individuals with germline *RB1 *variants face an increased risk of developing second primary malignancies later in life [17]. As a result, ongoing surveillance beyond childhood is necessary, further contributing to the long-term medical and psychological burden of heritable Rb.

Genetic testing is critical for all Rb patients as it distinguishes heritable from non-heritable disease and guides appropriate management. Identifying a germline *RB1 *variant in a proband enables targeted predictive testing for at-risk family members, including siblings and future offspring, to identify which individuals require the intensive surveillance protocol. Equally, it allows non-carrier relatives to be spared unnecessary EUAs and long-term follow-up, thereby reducing both familial burden and pressure on the healthcare system [16].

*RB1* gene testing routinely involves direct sequencing of the coding regions alongside deletion/duplication analysis, with the latter accounting for approximately 15%−25% of the causal variants in Rb cases [18, 19]. The large size of the *RB1 *gene and the widely distributed variants often make the molecular genetic diagnosis of Rb a challenge [20]. However, next-generation sequencing (NGS), with its capacity to analyze hundreds of samples simultaneously and sequence multiple genes in parallel, offers a highly efficient and powerful approach for both research and clinical diagnosis of Rb [21].

Despite over 80% of global Rb cases occurring in LMICs, there is limited attention and published literature on disease characteristics in these populations. Data regarding African Rb studies are particularly scarce and have largely focused on clinical presentation [22, 23]. A recent scoping review further highlighted the lack of genetic and molecular research in African populations and underscored the need to strengthen local capacity to improve patient outcomes [24]. Studying the *RB1* variant landscape in genetically diverse populations could unearth interesting variants with effects not seen previously and offer novel insights into Rb pathogenicity. Furthermore, understanding the genetic basis of Rb is crucial for improving patient management, as molecular diagnosis enables early detection, risk prediction, and informed clinical decisions. Therefore, this study aimed to explore the germline variant spectrum of the *RB1* gene in South African patients with clinically presumed heritable Rb, towards facilitating the implementation of a local genetic diagnostic service. A comprehensive approach was employed, combining NGS of coding regions and flanking splice sites with methylation-specific multiplex ligation-dependent probe amplification (MS-MLPA) to assess copy number variations and methylation profiles.

Establishing a genetic diagnostic service in SA would enable accurate risk prediction and early surveillance for the relatives of individuals with heritable Rb, including their future offspring through prenatal or early postnatal detection. This would support timely intervention for those at risk, and, importantly, reduce the need for frequent examinations under anaesthesia in individuals without a germline pathogenic variant, alleviating both the financial and emotional burden on families while easing the strain on a resource-constrained healthcare system.

## Methods

### Study cohort

Fifty-eight unrelated Rb patients, representing 49 families and nine singleton cases, were recruited from two tertiary academic hospitals in South Africa, between 2022 and 2024 (Table 1). The inclusion criteria were: (1) a family history of Rb (defined as at least one additional case among first- or second-degree relatives); (2) bilateral disease; or (3) unilateral disease with multifocal tumours. Criteria (2) and (3) were considered clinically presumed heritable Rb. Of the 58 patients, 50 (86.2%) presented with bilateral disease and eight with unilateral disease (13.8%). Among the unilateral cases, four had a family history of Rb, and five had multifocal tumors. Both affected minors and adults that met the criteria were eligible for enrolment and consent was obtained from each participant during recruitment, according to the tenets of the 2013 Declaration of Helsinki [25]. In the case of minors under the age of 16, informed consent to participate was obtained from the parents or legal guardians. The study was approved by the University of Cape Town (UCT) Human Research Ethics Committee (HREC), (HREC REF 203/2022).

**Table 1:** Demographic details of the study cohort

	Study Cohort (*n* = 58)
**Demographics**	
Age at diagnosis (months), mean ± SD (range)	13.9 ± 20.0 (0–132)
**Sex**,** n (%)**	
Male	33 (56.9%)
Female	25 (43.1%)
**Ethnicity**,** n (%)**	
Indigenous Black African	33 (56.9%)
Caucasian	7 (12.1%)
Mixed Ancestry	16 (27.6%)
Indian	2 (3.4%)

### DNA extraction and sample preparation

Blood and/or saliva samples were collected from each patient (and wherever possible from both biological parents and any other affected family members, *n* = 60 relatives) by the attending physician or registered nurse. Genomic DNA was extracted from blood samples using a modified version of the salting-out method [26], and from saliva samples using an Oragene^®^ saliva kit (DNA Genotek, Ottawa, Ontario, Canada) according to the manufacturer’s protocols. DNA concentration and integrity were assessed using the Nanodrop ND1000 Spectrophotometer (Thermo Fisher Scientific, Waltham, Massachusetts, United States), the Qubit dsDNA HS Assay Kit (Thermo Fisher Scientific), the TaqMan RNase P assay (Thermo Fisher Scientific), and agarose gel electrophoresis.

### Library preparation and next-generation sequencing

A NGS gene panel targeting the exons and exon/intron boundaries of 124 inherited retinal disease (IRD) genes, including *RB1*, was utilised [27]. Briefly, library preparation was performed using the Ion AmpliSeq Kit for Chef DL8 (Thermo Fisher Scientific) and subsequent quantification was performed using the Ion Library TaqMan Quantitation Kit (Thermo Fisher Scientific) on the CFX96 Real-Time System (Bio-Rad Laboratories, Hercules, California, United States), according to the manufacturer’s protocols. Templating and sequencing were performed using the Ion Chef Kit and instrument (Thermo Fisher Scientific), generating enriched ion sphere particles (ISPs). These were loaded onto an Ion 540 Chip and sequenced on the Ion S5™ (Thermo Fisher Scientific), a semiconductor based NGS system.

The Torrent Mapping Alignment Program aligned the sequencing data to the hg19 human reference genome. Following this, Binary Alignment/Map (BAM) files were processed through a custom workflow on the Ion Reporter Software (Thermo Fisher Scientific). Automated variant calling and annotation generated a variant call file (VCF) and quality control metrics.

### Single-nucleotide variant prioritization and classification

Two lists were generated to aid variant interpretation: an elimination list of presumed benign *RB1* variants and a pre-screen/priority list of known pathogenic and/or likely pathogenic *RB1* variants (Table S1 and S2). The elimination list was derived from (1) the Genome Aggregation Database (gnomAD) dataset, *gnomAD v2.1.1 (non-cancer) - gnomAD SVs v2.1* (accessed 16/03/2022), and (2) an in-house control dataset (*n *= 80) of non-Rb IRD patients previously screened using the IRD NGS gene panel [27]. All *RB1* gene variants detected in these two datasets were presumed to be benign. In parallel, the pre-screen list was compiled using variant data from the disease-specific databases ClinVar [28] (accessed 06/05/2022) and the Leiden Open Variation Database (LOVD) [11] (accessed 17/04/2022).

The *RB1* gene yielded an average of nine variants per proband sample. Initial filtering involved applying the elimination list to exclude presumed benign *RB1* variants. The remaining variants were then cross-referenced against the pre-screen list to identify any known pathogenic and/or likely pathogenic variants, which were prioritized for manual review. If no matches were identified, all remaining *RB1* variants underwent manual curation. When a clear causal pathogenic *RB1* variant was not identified, any overlapping variants from the elimination list were re-evaluated via manual curation.

Manual curation involved an evaluation of read depth, strand bias, and variant frequency within the cohort, using Integrative Genomics Viewer (IGV) to confirm that the variant was not an artifact [29]. Missense variants required a pathogenic prediction by at least three of the five pathogenicity annotations included in the Ion Reporter Software, i.e. SIFT, PolyPhen2, Grantham, FATHMM and PhyloP, to be further investigated [30–34]. All splice site and intronic variants, excluding those affecting the canonical splice acceptor (+ 1, + 2) and donor (− 1, − 2) positions, were evaluated using SpliceAI [35]. A predicted delta score ≥ 0.2 in any of the four categories (acceptor loss, donor loss, acceptor gain, donor gain) was considered indicative of pathogenicity. Subsequently, exonic splicing enhancer (ESE) analysis was performed using Alamut Visual Plus (v1.12) (SOPHiA GENETICS, Lausanne, Switzerland). Nonsense variants were assessed using Mutalyzer to evaluate the premature termination of protein translation, with a focus on the length and location of the predicted protein truncation [36]. Finally, the variants were classified according to the American College of Medical Genetics and Genomics (ACMG) guidelines for pathogenicity interpretation, with additional classification support from the online interpretation tool, Franklin (https://franklin.genoox.com) [37].

### Polymerase chain reaction and Sanger sequencing

All pathogenic and likely pathogenic single nucleotide variants (SNVs) or small insertions and deletions (indels) were validated by Sanger sequencing and screened in familial samples where possible. Primers were designed to cover whole exons of the *RB1 *gene using online software tools including Ensembl [38], Primer3Plus [39, 40], National Center for Biotechnology Information (NCBI) Primer- BLAST [41], and UCSC In Silico PCR (Table S3) [42]. Polymerase chain reaction (PCR) conditions were optimized for each *RB1* primer set by adjusting cycling conditions, annealing temperature, DNA template quantity, and magnesium chloride (MgCl_2_) concentration. In general, the thermocycling parameters consisted of an initial denaturation step at 95 °C for 5 min, followed by 30 cycles of successive denaturation at 95 °C for 30 s, an annealing step for 30 s, and extension at 72 °C for 40 s. The reaction concluded with a final extension step at 72 °C for 7 min. PCRs were conducted on a SimpliAmp Thermal Cycler (Thermo Fisher Scientific) according to the optimised conditions for each primer set.

Sanger sequencing was performed on an Applied Biosystems 2720 Thermal Cycler (Thermo Fisher Scientific) for an initial denaturation step at 96 °C for 5 min, followed by 30 cycles at 96 °C for 30 s, 50 °C for 15 s, and 60 °C for 4 min. In general, each sequencing reaction consisted of 100–300 ng PCR purified PCR product, 10 pmol primer, 1 X BigDye^®^ Terminator v3.1 buffer (Applied Biosystems, Thermo Fisher Scientific) and 1 X BigDye^®^ Terminator v3.1 Cycle Sequencing mix (Applied Biosystems). Following post-sequencing ethanol precipitation, the sequencing products were subjected to capillary electrophoresis on a SeqStudio Genetic Analyzer (Thermo Fisher Scientific). BioEdit Sequence Alignment Editor v7.2.534 was used to align the sequences to the *RB1 *reference sequence (NM_000321.3) obtained from Ensembl Genome Browser [38], while Chromas v2.6.6 (Technelysium Pty Ltd) was used to analyze the electropherograms.

### Next-generation sequencing analysis of copy number variants

To investigate large deletions and duplications in the *RB1* gene, a copy number baseline workflow preset was created on the Ion Reporter Software (Thermo Fischer Scientific). The copy number variant (CNV) baseline was generated using non Rb IRD samples (*n* = 74) with a mean sequencing depth >100X and uniformity >90%, according to the manufacturer’s protocols. The CNV baseline was subsequently incorporated into the Ion Reporter analysis workflow for CNV detection. NGS read depth in the proband samples could be accurately compared against the baseline to detect copy number changes across the *RB1* gene.

All CNV calls generated by Ion Reporter were first subjected to quality filtering to differentiate genuine CNV events from false-positive CNV calls. CNVs required a confidence score >20 and a precision score >10 to proceed to downstream evaluation. As the CNVs were generated by a baseline algorithm rather than a cytogenetic assay, the precise breakpoints, orientation, and size were not determined, limiting the immediate application of the ACMG and the Clinical Genome Resource (ClinGen) CNV classification guidelines. Therefore, putative CNVs that passed quality control filtering were subsequently validated using MS-MLPA, and only validated CNVs were classified according to the ACMG/ClinGen framework.

### Methylation-specific multiplex ligation-dependent probe amplification

MS-MLPA analysis was performed using the SALSA MLPA kit PO47-E2 RB1 (MRC-Holland, Amsterdam, the Netherlands) according to the manufacturer’s protocol and was used to validate all putative pathogenic and/or likely pathogenic CNVs that passed Ion Reporter quality control filtering. Additionally, in our workflow, any sample that remained unresolved after NGS and Sanger sequencing was further analyzed with MS-MLPA regardless of whether a CNV had been flagged by the baseline algorithm. This approach ensured comprehensive detection of *RB1* deletions and duplications and allowed confirmation of CNV calls prior to ACMG/ClinGen classification.

The SALSA MLPA kit P047-E2 RB1 includes 57 probes, with 35 probes covering all *RB1* exons (excluding exon 15). Additionally, five probes target the *RB1* promoter (CpG106), and three probes target the imprinted CpG island (CpG85) in intron two. MS-MLPA probes containing *Hha*I recognition sites provide methylation status at specific GCGC sites within CpG106 and CpG85. The kit also includes 13 reference probes targeting copy number-stable regions across various cancers, which remain unaffected by *Hha*I digestion. A dedicated digestion control probe confirmed the successful completion of restriction enzyme digestion in the MS-MLPA reactions.

Briefly, 100 ng of genomic DNA in 5 µL was denatured at 98 °C for 5 min, followed by incubation at 95 °C for 1 min and hybridization at 60 °C for 18 h. Ligase buffer A (13 µL) was added, and the mixture was separated into two tubes. For the copy number test, 10 µL ligase-65 mix was added, while the methylation test received 10 µL ligase-digestion mix. The samples were then incubated at 48 °C for 30 min. The ligase enzyme was inactivated by heating at 98 °C for 5 min. The PCR reaction included 5 µL polymerase mix, and thermocycling parameters involved 35 cycles of denaturation, annealing, and extension, followed by a final incubation at 72 °C for 20 min. The PCR products were then run on a 3500 Genetic Analyzer (Applied Biosystems), and the sequencing products were separated according to size.

### Cost comparison of retinoblastoma genetic testing versus conventional clinical screening

SA’s healthcare system is characterized by a two-tier structure marked by significant inequality. The public healthcare sector, which is funded by the government, serves approximately 71% of the population. In contrast, the private healthcare sector, primarily financed through personal medical aid contributions or health insurance, caters to about 27% of the population. The public sector suffers from underfunding, while the high costs of private healthcare remain unaffordable for most South Africans [43].

Hereditary familial Rb was confirmed in seven families (Figure S1), and the RB 29 family pedigree was used as an example to compare the costs of genetic testing with those of conventional clinical screening and to illustrate potential cost savings at the family level (Table S4). Conventional clinical screening was defined as an initial consultation and 22 EUAs up to the age of seven (as per Freeman and Meyer [16]), followed by annual in-room examinations with fundus photography until age 80. Associated transport and accommodation costs were also included, and total costs were calculated for both public and private healthcare sectors in SA.

The cost of genetic testing encompasses (1) testing of the proband and (2) cascade testing, defined here as genetic testing of at-risk offspring of affected individuals. This definition reflects a proband-led, descendant-focused cascade testing strategy and therefore does not necessitate inclusion of parental testing in the cost model. All costs are presented in 2025 United States Dollars (USD) and South African Rand (ZAR), using an average 2025 exchange rate of 1 USD = 18.48 ZAR.

To evaluate the potential long-term impact of genetic testing on cost-saving, we modelled a hypothetical expansion of the family into a subsequent generation and incorporated these estimates into the calculations presented in Supplementary Table 4. Using a 50% transmission probability for individuals in Generation IV, and applying South Africa’s 2023 fertility rate of 2.2 (The World Bank Group), each individual was assumed to have two children, and the family was projected to have 10 children in the next generation. Under these assumptions, the two hypothetical children of IV:7 were expected to be unaffected, while the remaining eight, born to affected parents, each had a 50% chance of inheriting the pathogenic *RB1* variant and thus would require genetic testing. This resulted in an estimated four affected and six unaffected children in the hypothetical generation.

## Results

### Age at diagnosis

The age at diagnosis for the 58 Rb cases ranged from 0 to 132 months, with a mean of 13.9 ± 20.0 months. This is lower than the mean age of diagnosis reported in the South African National Cancer Registry, i.e. 30 months [4]. Notably, our study observed an excess of bilateral disease, which is attributed to our focus on clinically presumed heritable cases and our recruitment sites. Bilateral (clinically presumed heritable) cases tend to be diagnosed at a younger age compared to unilateral (sporadic) cases. The large standard deviation reflects considerable variability, with some cases detected at or shortly after birth, while others were diagnosed as late as 11 years. This variability may have been influenced by differences in disease presentation and, most likely, access to healthcare facilities across SA.

### Benign genetic variation in the *RB1* gene

**Fig. 1:**
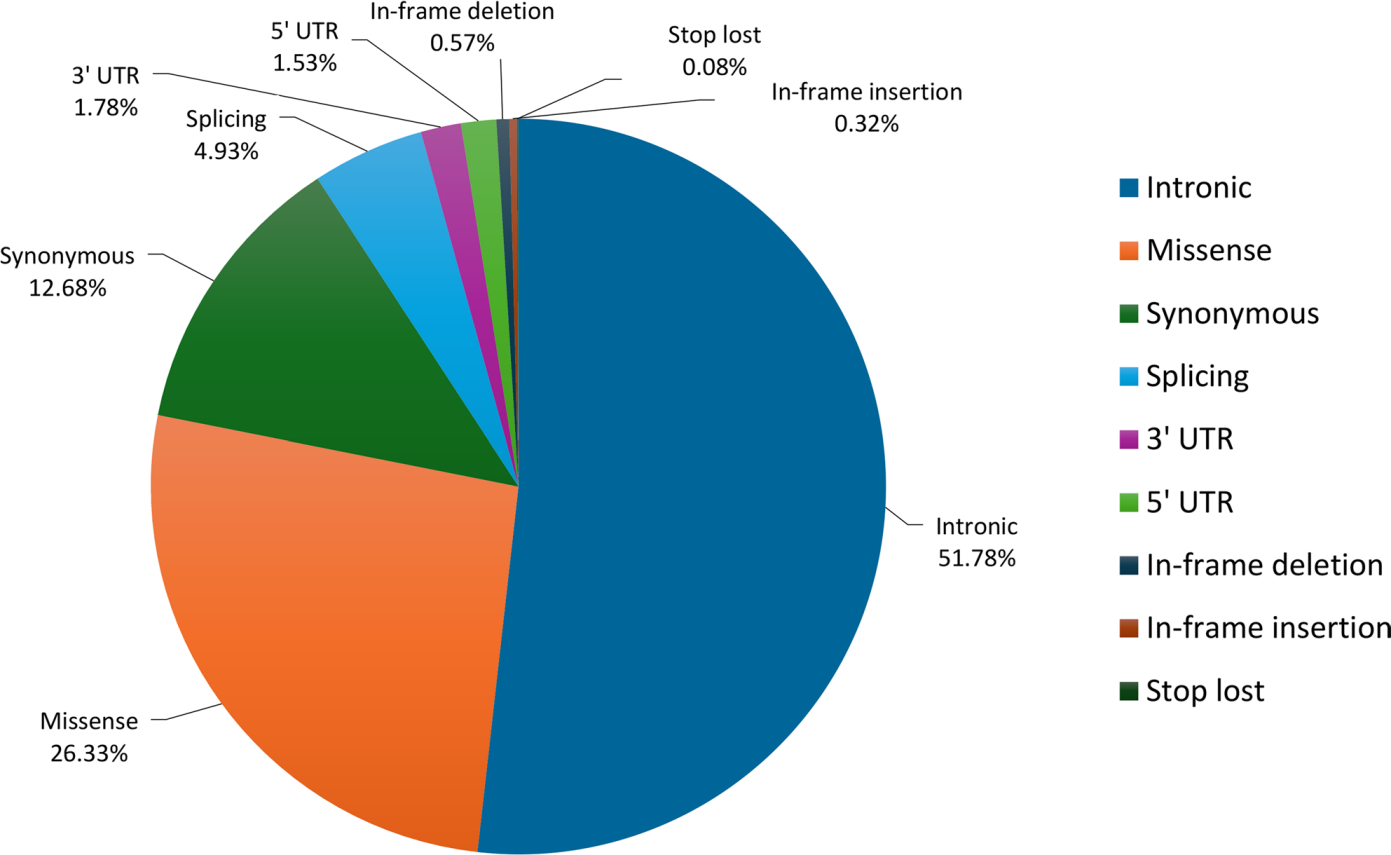
Distribution of the variant subtypes in the *RB1* gene included in the gnomAD non-cancer dataset. Below each variant subtype is the percentage of that subtype in the dataset. UTR untranslated region

The gnomAD dataset for the *RB1* gene (*gnomAD v2.1.1 (Non-cancer) - gnomAD SVs v2.1*; downloaded 16/03/2022) contained 1238 variants (Fig. 1). Of these, 51.78% (641/1238) were classified as intronic, 26.33% (326/1238) as missense, and 12.68% (157/1238) as synonymous, with the remaining variant subtypes contributing less significantly (Fig. 1). Subsequent inspection of the dataset focused on variants with an allele count of one (i.e., unique variants) and variants with an allele count greater than one (i.e., recurrent variants). Intronic variants were found to be the most prevalent subtype in both groupings.

Following this initial analysis, the gnomAD variant data for the African/African-American population was examined more closely. Within this subgroup, 64.14% (195/304) of the variants were classified as intronic, 19.74% (60/304) as missense, and 8.22% (25/304) as synonymous, with the remaining variant subtypes contributing less significantly. This distribution closely mirrors the global pattern of *RB1* variant subtypes shown in Fig. 1, with intronic variants being the most prevalent in both recurrent and non-recurrent groupings.

**Fig. 2:**
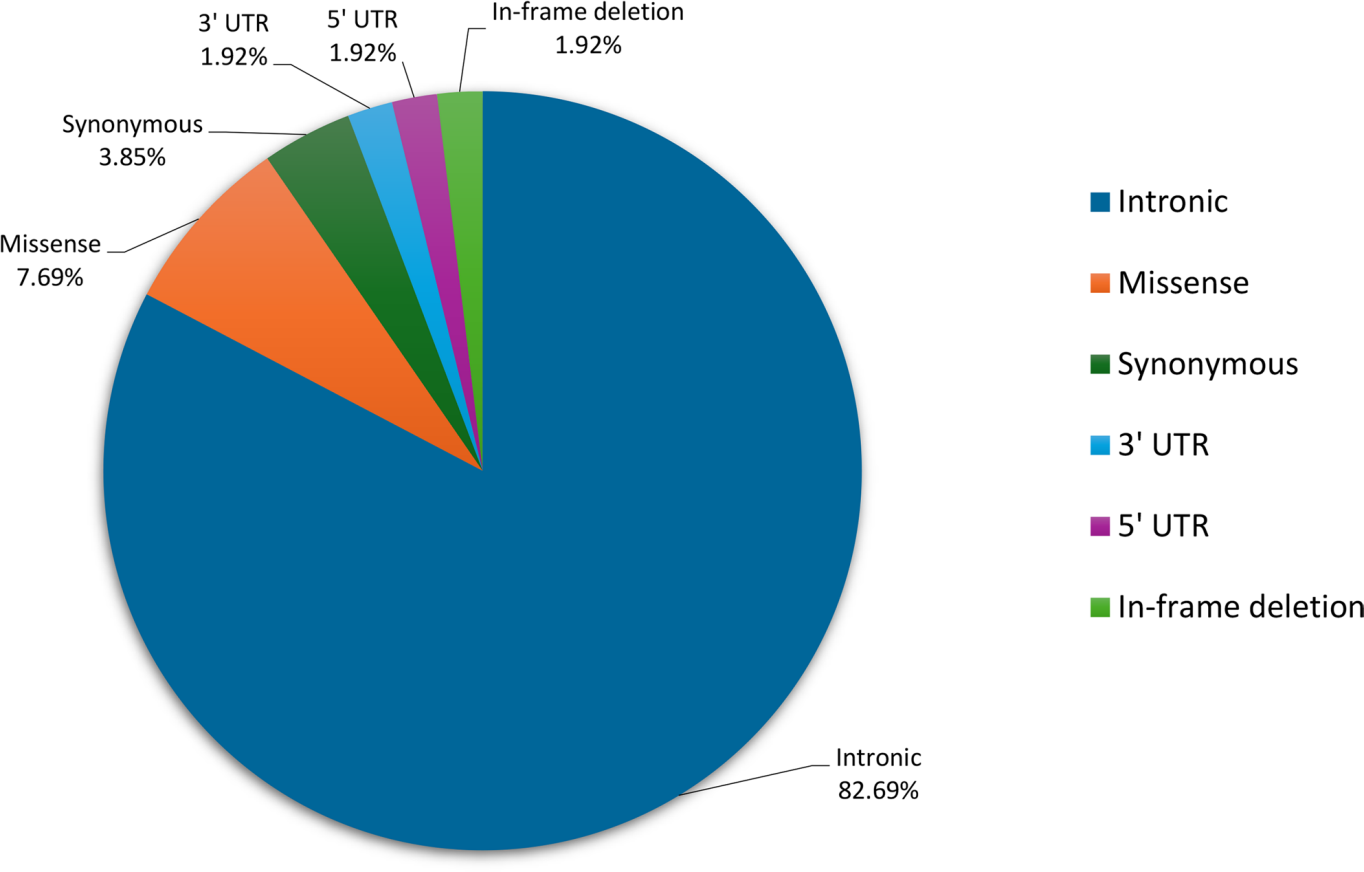
Distribution of the variant subtypes in the *RB1* gene identified in the in-house control dataset (n = 80). Below each variant subtype is the percentage of that subtype in the dataset. UTR untranslated region

To supplement the gnomAD data, *RB1* genetic data from an in-house control dataset, of 80 individuals with retinal diseases other than Rb, was analyzed. A total of 52 unique, presumed benign *RB1* SNVs and 20 unique, presumed benign *RB1* CNVs were identified (Table S1 and Table S5). Of the 52 SNVs, 25 were recurrent and 27 were observed once. Intronic variants made up the majority (82.69%), followed by missense (7.69%) and synonymous (3.85%) variants, with other variant subtypes contributing minimally (Fig. 2). This distribution is consistent with the global pattern of benign *RB1* variant subtypes (Fig. 1).

### Next-generation sequencing and Ion Torrent software analysis

All 58 patients were screened using the custom-designed 124-gene IRD panel. On average, 5,119,513 reads were obtained per sample with a mean depth of 1466X. Furthermore, 98.15% of the target bases had a minimum depth of 100X. ISP loading averaged 95%, with 60% usable reads and 37% polyclonality exhibited.

The NGS gene panel delivered 100% in-silico coverage across the entirety of the *RB1* gene. This was accomplished with 97.4% gene uniformity, using 31 amplicons to effectively span all 3057 bases of the coding sequence of *RB1*.

On average, *RB1* amplicons achieved 98.9% coverage at 20X, 95.3% at 100X, and 73.7% at 500X, demonstrating the high sequencing depth of the IRD gene panel across the *RB1* gene (Figure S2). This level of coverage supported confident variant detection and comprehensive genetic assessment.

**Table 2:** Summary of causal constitutional *RB1* variants identified in Southern African patients with heritable Rb

Patient	Family history	Ethnicity	Sex	Laterality^a^	Diagnosis age^b^	Age of onset	Exon/ Intron	cDNA variant	Protein variant	Variant type	Reported	Mother (G; *P*)^c^	Father (G; *P*)^c^
RB 4.1	Yes	C	F	Unilateral	3.5 mo	3 mo	1	c.37_65del	p.(Ala13ThrfsTer8)	Frameshift	ClinVar rs1064792974	c.37_65del/WT; affected (bilateral)	WT/WT; unaffected (no ophthalmic exam)
RB 5.2	No	B	M	Bilateral	6 mo	4 mo	Intron 22	c.2212-13T>A	-	Intronic	ClinVar & LOVD rs2138344431	WT/WT; unaffected (no ophthalmic exam)	Unknown; unaffected (no ophthalmic exam)
RB 6.2	No	B	M	Bilateral	6 mo	2 mo	11	c.1060C>T	p.(Gln354Ter)	Nonsense	LOVD rs764547244	WT/WT; unaffected (no ophthalmic exam)	Unknown; unaffected (no ophthalmic exam)
RB 7.1	No	B	M	Bilateral	12 mo	11 mo	17	c.1589A>G	p.(Lys530Arg)	Missense	ClinVar & LOVD rs1948534047	c.1589 A > G/WT; unaffected (no ophthalmic exam)	Unknown; unaffected (no ophthalmic exam)
RB 8.1	No	MA	F	Bilateral	24 mo	24 mo	8	c.763C>T	p.(Arg255Ter)	Nonsense	ClinVar & LOVD rs587778842	WT/WT; unaffected (no ophthalmic exam)	Unknown; unaffected (no ophthalmic exam)
RB 9.1	No	MA	F	Bilateral	10.5 mo	10.5 mo	1–27			Monoallelic *RB1* whole gene deletion +MED4, ITM2B, RCBTB2, DLEU1 & PCDH8		WT/WT; unaffected (no ophthalmic exam)	Unknown; unaffected (no ophthalmic exam)
RB 10.1	No	B	F	Bilateral	3 mo	3 mo	3	c.363dup	p.(Lys122GlufsTer9)	Frameshift	Novel	WT/WT; unaffected (no ophthalmic exam)	Unknown; unaffected (no ophthalmic exam)
RB 11.1	Yes	C	M	Bilateral	2 mo	2 mo	13–14			Monoallelic *RB1* exon 13–14 deletion		Unknown; affected (bilateral)	Unknown; unaffected (no ophthalmic exam)
RB 13.2	No	B	M	Bilateral	4 mo	3 mo	11	c.1063A>T	p.(Arg355Ter)	Nonsense	Novel	Unknown; unaffected (no ophthalmic exam)	Unknown; unaffected (no ophthalmic exam)
RB 14.1	No	B	F	Bilateral	4 mo	3 mo	22	c.2212-1_2214del	-	Splicing	ClinVar rs2542375089	WT/WT; unaffected (no ophthalmic exam)	Unknown; unaffected (no ophthalmic exam)
RB 15.1	No	B	M	Unilateral	6 wk	5 wk	12	c.1215+1G>A	-	Splicing	ClinVar & LOVD rs587776783	WT/WT; unaffected (no ophthalmic exam)	Unknown; unaffected (no ophthalmic exam)
RB 16.1	Yes	B	F	Bilateral	6 mo	3 mo	8	c.751C>T	p.(Arg251Ter)	Nonsense	ClinVar & LOVD rs1131690863	Unknown; affected (bilateral)	Unknown; unaffected (no ophthalmic exam)
RB 17.2	Yes	B	M	Unilateral	3 wk	3 wk	14	c.1333C>T	p.(Arg445Ter)	Nonsense	ClinVar & LOVD rs3092891	WT/WT; unaffected (no ophthalmic exam)	Unknown; affected (bilateral)
RB 18.1	No	B	M	Bilateral	9 mo	2 wk	14	c.1363C>T^F^	p.(Arg455Ter)	Nonsense (germline mosaic)	ClinVar & LOVD rs121913302	WT/WT; unaffected (no ophthalmic exam)	Unknown; unaffected (no ophthalmic exam)
RB 19.2	No	B	F	Bilateral	8 mo	7 mo	17	c.1515dup	p.(Leu506SerfsTer2)	Frameshift	Novel	WT/WT; unaffected (no ophthalmic exam)	Unknown; unaffected (no ophthalmic exam)
RB 20.1	No	B	F	Bilateral	24 mo	Unk	18	c.1735C>T	p.(Arg579Ter)	Nonsense	ClinVar & LOVD rs121913305	WT/WT; unaffected (no ophthalmic exam)	Unknown; unaffected (no ophthalmic exam)
RB 21.1	Yes	B	M	Bilateral	22 mo	10 d	17	c.1544del	p.(Pro515HisfsTer4)	Frameshift	LOVD no rs ID	WT/WT; unaffected (no ophthalmic exam)	Unknown, affected (bilateral)
RB 24.3	No	MA	M	Bilateral	3 mo	2.5 mo	1–27			Monoallelic RB1 whole gene deletion +MED4, ITM2B, RCBTB2, DLEU1 & PCDH8		WT/WT; unaffected (no ophthalmic exam)	Unknown; unaffected (no ophthalmic exam)
RB 25.2	No	B	M	Bilateral	9 mo	7 mo	12	c.1215+1G>A	-	Splicing	ClinVar & LOVD rs587776783	WT/WT; unaffected (no ophthalmic exam)	Unknown; unaffected (no ophthalmic exam)
RB 26.2	No	B	F	Bilateral	12 mo	6 mo	8	c.771del	p.(Asn258ThrfsTer6)	Frameshift	Novel	WT/WT; unaffected (no ophthalmic exam)	Unknown; unaffected (no ophthalmic exam)
RB 28.1	No	MA	M	Bilateral	3.5 mo	3 mo	4	c.381–2A>G	-	Splicing	ClinVar & LOVD rs1952480014	WT/WT; unaffected (no ophthalmic exam)	Unknown; unaffected (no ophthalmic exam)
RB 29.1^d^	Yes	MA	F	Bilateral	2.5 mo	2 mo	17	c.1572_1573del	p.(Lys524AsnfsTer3)	Frameshift	Novel	Unknown; unknown (no ophthalmic exam)	Unknown; unaffected (no ophthalmic exam)
RB 30.1	Yes	B	F	Bilateral	6.5 mo	6.5 mo	1	c.94del	p.(Asp32ThrfsTer33)	Frameshift	Novel	c.94del/WT; affected (unilateral)	Unknown; unaffected (no ophthalmic exam)
RB 31.1	Yes	MA	F	Bilateral	Birth	Birth	4	c.496G>T	p.(Glu166Ter)	Nonsense	ClinVar & LOVD rs1131690874	Unknown; affected (bilateral)	Unknown; unaffected (no ophthalmic exam)
RB 32.1	No	MA	M	Bilateral	12 mo	18 mo	12	c.1128-1G>A	-	Splicing	ClinVar & LOVD rs2138130852	WT/WT; unaffected (no ophthalmic exam)	Unknown; unaffected (no ophthalmic exam)
RB 33.1	No	MA	F	Bilateral	30 mo	4 mo	20	c.1961–2A>C	-	Splicing	Novel	WT/WT; unaffected (no ophthalmic exam)	Unknown; unaffected (no ophthalmic exam)
RB 34.1	Yes	B	M	Unilateral	2 mo	1 mo	23	c.2326–2A>G	-	Splicing	ClinVar & LOVD rs1949432057	c.2326–2 A > G/WT; affected (unilateral)	Unknown; unaffected (no ophthalmic exam)
RB 35.2	No	B	F	Bilateral	5 mo	3 mo	8	c.849del	p.(Cys283Ter)	Nonsense	Novel	WT/WT; unaffected (no ophthalmic exam)	Unknown; unaffected (no ophthalmic exam)
RB 36.1	No	B	M	Bilateral	24 mo	24 mo	23	c.2425del	p.(Leu809Ter)	Nonsense	LOVD no rsID	WT/WT; unaffected (no ophthalmic exam)	Unknown; unaffected (no ophthalmic exam)
RB 37.2	No	B	F	Bilateral	5 mo	5 mo	1	c.45_76del	p.(Ala17ProfsTer3)	Frameshift	ClinVar & LOVD rs1593412002	WT/WT; unaffected (no ophthalmic exam)	Unknown unaffected (no ophthalmic exam)
RB 38.2	No	B	F	Bilateral	25 mo	22 mo	4	c.409G>T	p.(Glu137Ter)	Nonsense	ClinVar & LOVD rs121913296	WT/WT; unaffected (no ophthalmic exam)	Unknown; unaffected (no ophthalmic exam)
RB 40.1	No	B	M	Bilateral	12 mo	12 mo	21	c.2139dup	p.(Val714SerfsTer7)	Frameshift	LOVD no rsID	Unknown; unaffected (no ophthalmic exam)	Unknown; unaffected (no ophthalmic exam)
RB 41.1	No	B	M	Bilateral	12 mo	12 mo	6	c.607+1G>A	-	Splicing	ClinVar & LOVD rs587776789	WT/WT; unaffected (no ophthalmic exam)	WT/WT; unaffected (no ophthalmic exam)
RB 42.1	No	B	F	Bilateral	6 mo	3 mo	12	c.1215+1G>A	-	Splicing	ClinVar & LOVD rs587776783	WT/WT; unaffected (no ophthalmic exam)	Unknown; unaffected (no ophthalmic exam)
RB 43.1	No	MA	F	Bilateral	23 mo	23 mo	10	c.958C>T	p.(Arg320Ter)	Nonsense	ClinVar & LOVD rs121913300	WT/WT; unaffected (no ophthalmic exam)	WT/WT; unaffected (no ophthalmic exam)
RB 44.1	No	B	F	Bilateral	3 mo	3 mo	1–27			Monoallelic RB1 whole gene deletion +MED4, ITM2B, RCBTB2, DLEU1 & PCDH8		Monoallelic RB1 whole gene deletion +MED4, ITM2B, RCBTB2, DLEU1 & PCDH8; unaffected (no ophthalmic exam)	Unknown; unaffected (no ophthalmic exam)
RB 45.1	No	MA	M	Bilateral	12 mo	7 mo	2	c.138-1G>T	-	Splicing	ClinVar rs1593414337	Unknown; unaffected (no ophthalmic exam)	Unknown; unaffected (no ophthalmic exam)
RB 47.1	No	MA	M	Bilateral	2.5 mo	1 mo	8	c.751C>T	p.(Arg251Ter)	Nonsense	ClinVar & LOVD rs1131690863	WT/WT; unaffected (no ophthalmic exam)	WT/WT; unaffected (no ophthalmic exam)
RB 49.1	No	I	F	Bilateral	15 mo	12 mo	20	c.2042G>A	p.(Trp681Ter)	Nonsense	ClinVar & LOVD rs2138336207	WT/WT; unaffected (no ophthalmic exam)	WT/WT; unaffected (no ophthalmic exam)
RB 50.1	No	C	M	Bilateral	4 mo	`4 mo	1–27			Monoallelic *RB1* whole gene deletion +ENOX1, MED4, ITM2B, RCBTB2, DLEU1 & PCDH8		WT/WT; unaffected (no ophthalmic exam)	WT/WT; unaffected (no ophthalmic exam)
RB 51.1	No	I	M	Bilateral	22 mo	15 mo	11	c.1064_1065del	p.(Arg355AsnfsTer6)	Frameshift	ClinVar & LOVD rs1131690861	WT/WT; unaffected (no ophthalmic exam)	WT/WT; unaffected (no ophthalmic exam)
RB 52.1	Yes	C	M	Bilateral	8 wk	Birth	20	c.1981del	p.(Arg661GlyfsTer2)	Frameshift	Novel	WT/WT; unaffected (no ophthalmic exam)	c.1981del/WT; affected (bilateral)
RB 55.1^e^	No	C	F	Bilateral	8.5 mo	2 mo	1	c.45_76del^F^	p.(Ala17ProfsTer3)	Frameshift	ClinVar & LOVD rs1593412002	Unknown; unaffected (no ophthalmic exam)	Unknown; unaffected (no ophthalmic exam)
RB 56.2	No	MA	M	Bilateral	18 mo	16 mo	18	c.1696-1_1696delinsCT	-	Splicing	Novel	WT/WT; unaffected (no ophthalmic exam)	Unknown; unaffected (no ophthalmic exam)
RB 59.1	No	B	F	Bilateral	12 mo	19 mo	1	c.83dup	p.(Pro29SerfsTer2)	Frameshift	ClinVar & LOVD no rsID	Unknown; unaffected (no ophthalmic exam)	Unknown; unaffected (no ophthalmic exam)
RB 60.1^d^	Yes	B	M	Bilateral	5 mo	5 mo	24–26			Monoallelic *RB1* exon 24–26 deletion		Unknown; unaffected (no ophthalmic exam)	Unknown; unaffected (no ophthalmic exam)
RB 61.1	Yes	B	F	Bilateral	3 mo	7 wk	23	c.2359C>T	p.(Arg787Ter)	Nonsense	ClinVar & LOVD rs137853293	c.2359 C > T/WT; affected (bilateral)	Unknown; unaffected (no ophthalmic exam)
RB 62.1	No	B	M	Bilateral	15 mo	10 mo	19	c.1939_1940del	p.(Leu647PhefsTer5)	Frameshift	ClinVar & LOVD rs2138331562	WT/WT; unaffected (no ophthalmic exam)	WT/WT; unaffected (no ophthalmic exam)
RB 63.1	No	C	M	Bilateral	6 mo	3 mo	20	c.2054_2055insAA	p.(His686SerfsTer11)	Frameshift	Novel	WT/WT; unaffected (no ophthalmic exam)	WT/WT; unaffected (no ophthalmic exam)
RB 64.1	No	B	M	Bilateral	23 mo	15 mo	10	c.940–2A>T	-	Splicing	LOVD No rsID	WT/WT; unaffected (no ophthalmic exam)	Unknown; unaffected (no ophthalmic exam)
RB 65.1	No	B	F	Bilateral	4 mo	3 mo	14	c.1333C>T	p.(Arg445Ter)	Nonsense	ClinVar & LOVD rs3092891	WT/WT; unaffected (no ophthalmic exam)	Unknown; unaffected (no ophthalmic exam)

^a^Laterality refers to the clinical diagnosis at recruitment as determined by the treating ophthalmologist

^b^Diagnosis age refers to the age at which a retinoblastoma was first detected by the treating ophthalmologist

^c^If unknown biological samples were not available from the parent for testing

^d^Probands children are affected

^e^No Sanger sequencing performed due to sample depletion

^F^Not included in the overall detection rate - pending orthogonal validation

*C* Caucasian, *B* Indigenous Black African, *MA* Mixed Ancestry, *F* Female, *M* Male, *G* Genotype, *P* Phenotype, *WT* Wild type

### Spectrum of *RB1* variants identified in cases

A total of 58 South African patients with Rb [33 males (56.9%) and 25 females (43.1%)], were recruited for genetic testing of germline variants in the *RB1* gene. Parental testing was also performed, where possible, for families in which a pathogenic or likely pathogenic *RB1* variant was identified. Of the 58 patients, 50 (86.2%) presented with bilateral disease and eight with unilateral disease (13.8%). Using the NGS panel, germline *RB1* variants were identified in 43 of the 58 patients (74.1%) (Table 2), comprising SNVs and indels. Two cases (RB 18.1 and RB 55.1), flagged in Table 2, were excluded from the overall detection rate pending orthogonal validation. The NGS gene panel also facilitated the detection of CNVs through an embedded CNV baseline, which were subsequently validated by MS-MLPA. All CNVs detected by MS-MLPA had been previously called in the NGS data, with no missed cases. In total, CNVs were identified in six probands (6/58, 10.3%), increasing the resolution rate to 84.5%.

**Fig. 3:**
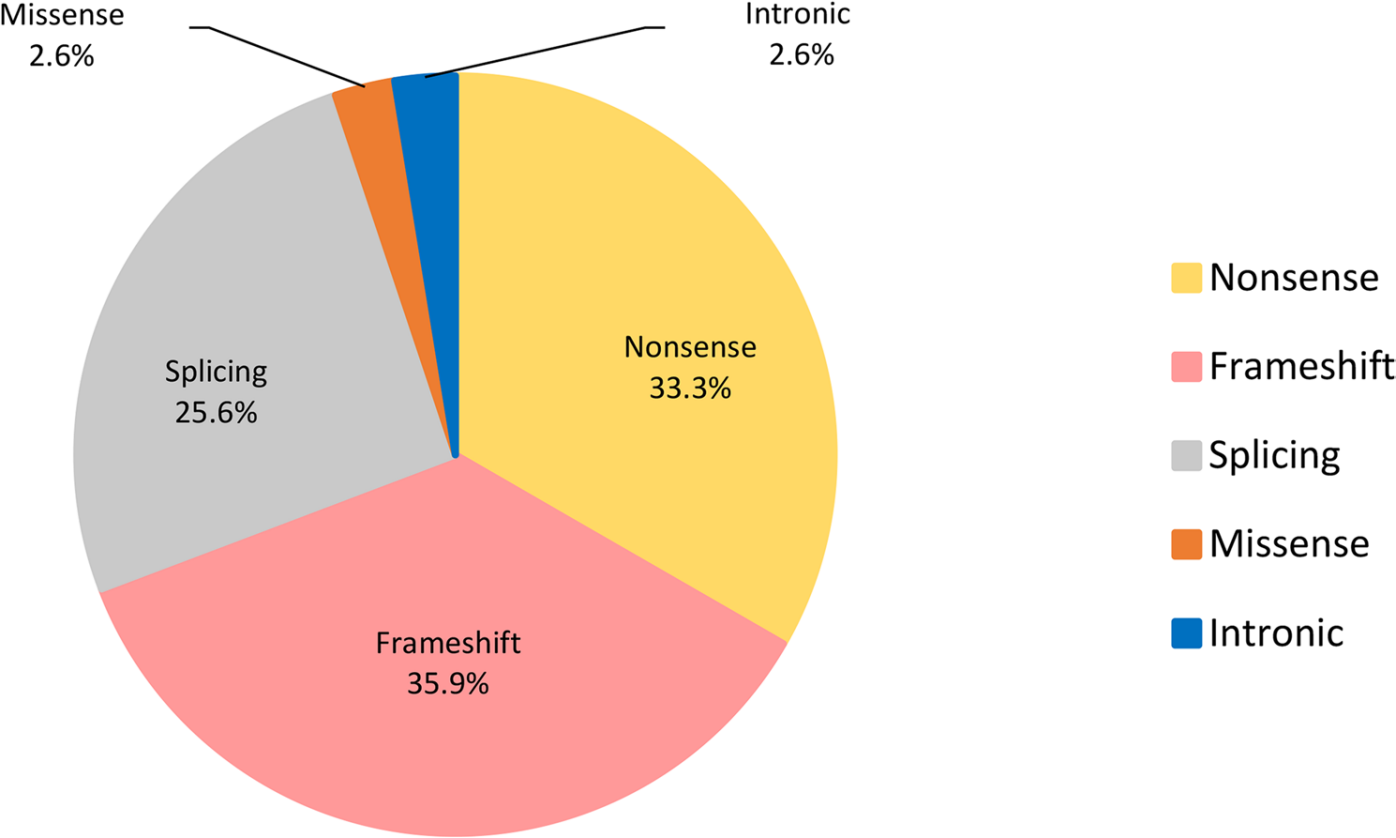
Distribution of the *RB1* single nucleotide variants and small insertions and deletions detected in this study. Below each variant subtype is the percentage of that subtype in the dataset

The spectrum of variant types among the 39 unique variants were: 35.9% (14/39) frameshift variants, followed by 33.3% nonsense (13/39), 25.6% (10/39) splicing, 2.6% (1/39) intronic and 2.6% (1/39) missense variants (Fig. 3 and Table S6). The nonsense variants primarily resulted from CGA to TGA transitions occurring within CpG dinucleotides. Seven of the 11 reported recurrent CGA>TGA nonsense *RB1* variants were detected in this Southern African cohort (Table 2 and Table S7) [2, 44]. Of these variants, c.751C>T in exon 8 and c.1333C>T in exon 14 were each identified in two unrelated individuals. NGS detected c.1363C>T at an alternative allele fraction of 11% (160/1392 reads) in blood DNA in patient RB 18.1, significantly lower than the 50% expected for a heterozygote (Figure S3A). This observation suggests mosaicism, further supported by the variant being undetectable by Sanger sequencing (Figure S3B). *RB1* c.1363C>T has been previously reported in the setting of hereditary Rb, is classified as pathogenic by numerous clinical laboratories (ClinVar ID: 126837), and is consistent with the diagnosis of Rb.

Frameshift and splicing variants emerged as the second most prevalent variant types, with half (7/14) of the frameshift variants being novel (defined here as not reported in ClinVar or the LOVD) (Figure S4 and Table S6). The most frequent reported splice variant in patients, c.1215 + 1G>A (reported 64 times in the LOVD [11]), was detected in three unrelated individuals in this cohort, two with bilateral disease and one with unilateral disease. This variant leads to skipping of exon 12, causing a frameshift and the introduction of a premature stop codon, which results in a truncated and non-functional protein [45].

Two unrelated patients with bilateral disease were found to carry a missense variant (c.1589A>G) and an intronic variant (c.2212-13T>A), respectively. The c.1589A>G variant was predicted to be deleterious by SIFT and probably damaging by PolyPhen-2 and was assigned a high conservation score in PhyloP (8.94), indicating strong evolutionary constraint at this position. Additionally, the meta-predictor REVEL assigned a high score (0.93), supporting a deleterious effect of the variant [37, 46]. c.1589A>G shows significantly increased prevalence in affected individuals compared to controls (ClinVar ID: 1037460) and is absent from large population databases, including the Exome Aggregation Consortium (ExAC) and gnomAD. Furthermore, the patient presented with bilateral disease and multifocal tumours, which is highly specific for Rb. Together these findings supported the classification of this variant as pathogenic. On the other hand, c.2212-13T>A was predicted by SpliceAI [35] to impact splicing, with acceptor loss and acceptor gain scores of 0.98 and 1.00, respectively. This variant similarly shows significantly increased prevalence in affected individuals compared to controls (ClinVar ID: 126799) and is absent from ExAC and gnomAD. Furthermore, the patient presented with bilateral disease and multifocal tumours. Together these findings supported the classification of this variant as pathogenic.

Several challenges arose in the analysis of two deletions in exon 1 of the *RB1* gene: c.37_65del and c.45_76del. In sample RB 4.1, NGS initially identified the c.37_65del variant as homozygous, which was biologically implausible. This apparent homozygosity was confirmed by Sanger sequencing using primers spanning exon 1 of the *RB1*gene, but preferential amplification was suspected. The use of alternative primers positioned closer to the variant, together with the addition of betaine to improve amplification of the highly GC-rich exon (Table S3 and Table S8) [47], ultimately revealed the variant to be heterozygous (Figure S5). Similarly, in sample RB 37.2, the c.45_76del variant was detected in only three NGS reads but gave an apparent homozygous result on Sanger sequencing. Applying the same modifications as above revealed the true nature of the variant to be heterozygous.

Gross deletions were detected in 10.3% of probands (Figure S6). In four of these cases the deletion encompassed the entire *RB1* gene, as well as neighboring genes; however, precise breakpoints were not determined. A deletion spanning *RB1* and extending from *MED4* to *PCDH8* was observed in cases RB 9.1, RB 24.3, and RB 44.1. The deletion in RB 44.1 was inherited from the Rb-unaffected mother (Figure S1E). For RB 9.1 and RB 24.3, parental analysis was not possible, and it remains unknown whether these deletions were inherited or arose de novo; however, in all cases hypermethylation of CpG85 suggests loss of the paternal allele. MS-MLPA analysis also revealed a gross deletion encompassing *RB1* and neighboring genes (*ENOX1*,* MED4*,* ITM2B*,* RCBTB2*,* DLEU1* and *PCDH8*) in patient RB 50.1. This deletion was identified as de novo and of the paternal chromosome. The remaining two gross deletions, identified in bilateral cases RB 11.1 and RB 60.1, were intragenic; a monoallelic deletion of exons 13–14 and exons 24–26, respectively. Additionally, RB 12.4 exhibited hypermethylation of CpG85; however, methylation analysis of the parental samples was inconclusive, preventing confirmation of whether this altered methylation profile was inherited.

### Cost comparison of conventional clinical screening and genetic testing for retinoblastoma

The total cost of conventional clinical screening for a single individual was estimated at US$32,803 in the private sector and US$23,874 in the public sector. In comparison, the cost of genetic testing for the proband was US$316 when using a blood sample, and US$333 when using a saliva sample. Cascade testing was considerably more affordable at US$24 when using a blood sample and US$41 when using a saliva sample. All amounts reflect the unit costs in our research laboratory and are specific to our workflow and institutional pricing (Table S4).

We assessed potential cumulative cost savings at the family level by comparing the standard approach of conventional clinical screening with a genetically guided approach, in which individuals without the pathogenic variant are spared unnecessary EUAs and lifelong surveillance. For the proband and Generation IV, the genetically guided approach resulted in cost savings between US$32,265 and US$32,365 in the private sector, and between US$23,336 and US$23,437 in the public sector. When incorporating a hypothetical fifth generation, these savings increased to between US$228,754 and US$228,989 in the private sector, and between US$166,254 and US$166,489 in the public sector (Table S4).

## Discussion

To the best of our knowledge no *RB1* genetic analyses have been reported in SA to date, and this study represents the most extensive analysis of germline *RB1* variants among Southern African patients with clinically presumed heritable Rb. Cataloging the variant spectrum of the *RB1* gene, including benign genetic variation, is crucial in establishing a robust genetic diagnostic service. Genetic testing is particularly valuable in heritable Rb, as it enables the precise identification of family members who carry the *RB1* pathogenic variant. This allows for the prioritization of at-risk relatives for intensive clinical screening to ensure early detection and containment of the disease, while sparing non-carriers from unnecessary EUAs and associated stress. Therefore, this study aimed to explore the germline variant spectrum of the *RB1* gene in Southern African patients with clinically presumed heritable Rb towards facilitating the implementation of a genetic diagnostic service for future integration into the South African healthcare system.

The analysis of benign genetic variation across the *RB1* gene revealed that most benign variants were intronic (Figs. 1 and 2), consistent with the permissive nature of non-coding regions. Three variants: c.2663+33C>T, c.500+23T>G, and c.1128-72G>T were detected at extremely high frequencies in both our in-house control dataset and the gnomAD dataset, with allele frequencies exceeding 93%. Notably, the gnomAD dataset includes data from various population groupings and is not ancestry-matched to our cohort, yet these high frequencies across diverse datasets suggest that the variants are likely to represent the major alleles in the general population. This highlights a limitation of the current human reference genome, which is largely derived from a single individual and does not adequately capture global genetic diversity [48]. In order to assist with our variant interpretation, the genetic variants from both control datasets were combined into a comprehensive elimination list (*n* = 1284), representing all presumed benign variants within the *RB1* gene (Table S1).

Our analysis of 58 patients with clinically presumed heritable Rb using NGS and MS-MLPA resolved the causative variant in 84.5% of patients. These results are similar to other studies, with variants detected in 80–85% of bilateral patients [49–51]. Analysis of the variant types revealed that the variant spectrum of the *RB1 *gene in Southern African patients closely resembles that reported in international literature [12, 13, 20, 52–56]. However, our study identified eleven novel *RB1* variants accounting for 25.6% (11/43) of all germline SNVs and indels detected (Table S6). These included seven frameshift, two nonsense, and two splicing variants, further expanding the known *RB1* variant spectrum and providing valuable insights for genetic diagnostics and counselling. Notably, six of these novel variants (55%) were detected in unrelated patients of Indigenous Black African ancestry, highlighting the contribution of underrepresented populations to the discovery of new *RB1* variants and emphasizing the importance of increasing diversity in reference datasets.

**Fig. 4:**
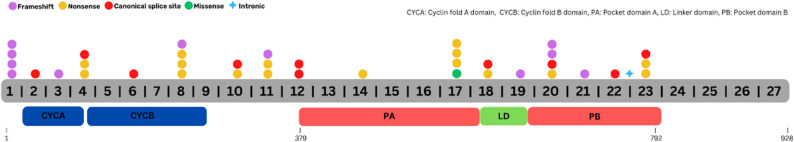
Schematic representation of the spectrum of causal *RB1* single nucleotide variants and small insertions and deletions identified. The cyclin fold domains (CYCA and CYCB) are highlighted in blue, the linker domain (LD) in green, and the pocket domains (PA and PB) in pink. Identical recurrent variants are represented once

pRb functions as a key tumor suppressor by regulating cell cycle progression, specifically controlling the G1-to-S phase transition. This regulation is mediated in part through its role as a transcriptional repressor of genes involved in cell cycle control. Central to this function is the conserved pocket domain, which serves as a molecular docking site for E2F transcription factors and other regulatory proteins, thereby ensuring precise control of the cell cycle and the prevention of uncontrolled cell proliferation. Structurally, the pocket domain is composed of two interdependent subdomains, A and B, which together form a functional motif essential for transcriptional repression [57]. Half (51.3%) of our identified variants were located within the pocket domain of pRb (exons 12–23), consistent with previous studies reporting similar distribution rates (65.4% [12], 58.6% [54], 58.1% [52], and 40% [55]). Among the variants detected in this region, 45% (9/20) fell within exons 12–18 (domain A), while 55% (11/20) fell within exons 19–23 (domain B) (Fig. 4). While variants were dispersed throughout the *RB1* gene, we identified recurrent pathogenic variants in exons 1, 8, 12, and 14.

Nonsense variants were the most prevalent subtype, primarily arising from CGA to TGA transitions at CpG dinucleotides. In the *RB1 *gene, arginine codons (CGA) frequently undergo this transition at 11 distinct positions in exons 8, 10, 11, 14, 15, 17, 18 and 23, accounting for nearly 30% of all reported point variants [2, 44]. Seven of these 11 recurrent variants were detected in this Southern African cohort, accounting for 16.3% (7/43) of the detected pathogenic SNVs and indels.

In our patient cohort, only one missense variant (c.1589A>G) [44, 58] and one intronic variant (c.2212-13T>A) [45] were identified, both previously reported as pathogenic and likely pathogenic in ClinVar, respectively. Missense variants in the *RB1 *gene typically localize to the pocket domain and are often associated with reduced penetrance, likely due to partial retention of pRb function; however, some carriers still develop bilateral disease and multifocal tumors [59, 60]. This is thought to be influenced by the variant’s specific position within the gene or the nature of the second hit [44, 61]. The c.1589A>G variant lies within domain A of the pocket domain of pRb, at a position that is highly conserved across species, as indicated by a high phyloP score (8.94). Should the c.1589A > G variant disrupt the structure or function of domain A, or interfere with its interaction with domain B, it is plausible that the capacity of pRb to effectively repress transcription could be compromised. A consequential loss of this repressor activity could contribute to the aberrant cell proliferation observed in tumor development. Interestingly, this missense variant was detected in the unaffected mother of the proband, suggesting incomplete penetrance or a modifying genetic background (Figure S1B). The c.2212-13T>A variant was predicted to disrupt the canonical splice acceptor site of exon 23 and activate a cryptic acceptor located two nucleotides downstream, resulting in the partial retention of 11 intronic nucleotides at the 5′ end of the exon. This aberrant splicing event introduces a frameshift that is expected to generate a premature stop codon, leading to loss of functional pRb. ESE motif analysis further revealed that the variant disrupts predicted binding sites for the splicing regulators SC35 and SRp40, both essential for proper 3′ splice site recognition. Their loss likely contributes to the aberrant splice site selection.

We found the 137-base pair exon 1 of *RB1 *(~ 75.9% GC content) to be particularly susceptible to preferential amplification and strand bias, complicating PCR and sequencing workflows and often necessitating specialized conditions [62, 63]. For both the c.37_65del and c.45_76del variants, pseudo-homozygosity was observed. Accurate amplification of both alleles required careful optimization including alternative primers and betaine supplementation which ultimately confirmed heterozygosity (Figure S5, Table S3, and Table S8). This underscores the importance of rigorous assay refinement when working with GC-rich regions to prevent the misinterpretation of zygosity.

Since gross deletions/duplications are detected in approximately 15% to 25% of familial (bilateral) Rb cases, CNV analysis was employed in this study [18, 19]. Using NGS data analysis and MS-MLPA confirmation, *RB1* deletions were identified in six of the 58 patients (10.3%) which increased the resolution rate to 84.5% (49/58). This included four whole-gene deletions and two intragenic deletions. Although large *RB1 *deletions can raise concern for 13q deletion syndrome [64], detailed phenotypic data for these patients were not collected at recruitment; however, during result delivery these individuals will be specifically assessed for additional features suggestive of 13q deletion syndrome through more comprehensive medical and family history taking. Notably, all four patients with monoallelic whole-gene *RB1* deletions also carried a deletion of the neighboring gene, *MED4*. *MED4*, a key component of the mediator complex, is essential for cell survival in the absence of *RB1*, earning it the designation of a ‘survival gene’. [65] Yet, these four patients exhibited bilateral disease, a phenotype distinct from the low-penetrance Rb typically associated with large *RB1* deletions that include *MED4*. The low penetrance is thought to result from cells not tolerating homozygous loss of *MED4*, thereby reducing the likelihood of tumor development [65]. However, MS-MLPA does not define precise deletion breakpoints, and breakpoint confirmation is required before inferring any mechanistic contribution of *MED4* loss to the observed phenotype.

Beyond copy number analysis, MS-MLPA also enabled assessment of the methylation status of the *RB1* promoter (CpG106) and the imprinted locus (CpG85) in intron 2. CpG85 is a differentially methylated CpG island derived from a 5’-truncated pseudogene of *KIAA0649 *on chromosome 9 [66]. On the maternal chromosome, CpG85 is hypermethylated, while on the paternal chromosome, it functions as a weak promoter for an alternative *RB1* transcript, *RB1-E2B* [66]. As a result, maternal *RB1 *allele loss leads to CpG85 hypomethylation, while paternal allele loss leads to CpG85 hypermethylation [67]. This was valuable in sample RB 44.1 which exhibited hypomethylation at CpG85, consistent with maternal inheritance of the deletion (Figure S6D). This deletion was inherited from the unaffected mother (Figure S1E). A limitation of this study was the incomplete screening of three familial cases (RB 12.1ADR, RB 12.3BAR, and RB 24.2KEE) due to failed methylation analyses. This was likely caused by technical issues such as poor DNA quality or enzyme inactivation, which underscores the need for more stringent sample quality control in future studies to ensure reliable methylation assessment.

Our findings demonstrate that genetic testing for Rb offers substantial cost savings compared to lifelong conventional clinical screening, particularly in families with a confirmed germline *RB1* pathogenic variant. These savings are especially significant when cascade testing is applied across multiple generations, highlighting the long-term economic benefit of integrating genetic testing into standard care. Importantly, our cost-saving model was based on a proband-led, descendant-focused cascade testing strategy, in which predictive testing is applied directly to siblings and offspring once the familial pathogenic *RB1* variant is identified. In our cohort, both parents were recruited for only 15 probands (approximately 26% of the total cohort), reflecting the practical realities of recruitment rather than a parent-dependent testing framework. While our cost calculations were based on a broad IRD panel currently in use in our research laboratory, it is worth noting that the cost of proband testing could be further reduced with a dedicated *RB1*-specific NGS panel. Such an approach would streamline analysis and reduce reagent costs, making genetic testing even more accessible and scalable in resource-constrained settings. In the context of SA’s dual-tiered healthcare system, where the majority rely on an under-resourced public sector, adopting a cost-effective, genetically guided approach could alleviate financial strain and reduce unnecessary procedures in unaffected individuals.

While genetic testing offers clear cost savings, it also addresses critical practical challenges in the South African context, where adherence to conventional lifelong clinical screening is variable. Barriers such as travel distance, financial constraints, and the stress of repeated EUAs can result in incomplete surveillance and delayed tumor detection. Integrating *RB1* genetic testing as a diagnostic service would not only improve risk stratification and reduce unnecessary procedures for non-carriers but also potentially enhance compliance with surveillance in those identified as at risk.

Targeted NGS also proved to be an effective tool for *RB1* variant detection in this cohort, identifying the causal variant in 74.1% of patients (43 of 58), despite the panel not being exclusively designed for Rb. The IRD gene panel is currently the standard workflow in our research laboratory and enables simultaneous screening of multiple retinal genes, including *RB1*, providing a cost-efficient approach in resource-limited settings. However, since Rb is predominantly a monogenic disease it is expected that the sensitivity of variant detection in *RB1 *should approach 100% when analyzing germline DNA in bilateral cases [68]. The presence of unresolved cases therefore suggests that some causal *RB1* variants remain undetected, potentially due to structural rearrangements (e.g. translocations), deep intronic variants (DIVs), or promoter variants not captured by our current assay. Although more than 97% of Rb cases result from *RB1* inactivation, a small minority (< 1%) are driven by *MYCN* oncogene amplification, a tumour-specific event occurring in patients with two normal copies of the *RB1 *gene [69]. These patients typically display an aggressive phenotype with early onset and fewer of the genomic copy number changes characteristic of Rb [70]. In addition, deleterious SNVs in *BCOR* and *CREBBP *represent the most frequent co-occurring somatic alterations in Rb tumours [71–73]. However, because *MYCN* amplification and variants in *BCOR* and *CREBBP* are strictly somatic and confined to tumour tissue, they cannot be detected in germline DNA and therefore do not explain the germline-negative unresolved cases in our cohort. It is more likely that these cases harbor undetected germline *RB1 *variants, including possible low-level mosaicism [74, 75]. Therefore, to improve diagnostic yield, unresolved cases could undergo long-read sequencing, which is capable of detecting complex structural variants often missed by conventional methods. This approach can also detect pathogenic alterations outside the coding regions, several of which have experimentally validated effects on splicing [18, 45, 50, 76–78].

## Conclusions

Beyond providing specific insights into Southern African Rb cases, these findings contribute to the global understanding of genetic variation within the *RB1* gene. This knowledge is crucial for advancing genetic diagnostic services and highlights the need for continued research to guide clinical decision-making, improve testing accessibility, and provide families with more accurate risk assessments. Importantly, we have shown that the introduction of genetic testing has significant implications for healthcare resource optimization, particularly in resource-constrained settings like SA. The associated cost savings of reducing unnecessary surveillance enable more efficient allocation of healthcare resources. For example, identifying non-carriers eliminates the need for routine EUAs and annual fundus photography, freeing up operating theatre time for higher-priority procedures and enabling more efficient allocation of healthcare resources. In addition to financial benefits, genetic testing and counselling also increase knowledge of carrier status, which may improve compliance with surveillance protocols among at-risk individuals. Non-carriers further benefit from being spared the physical burden, emotional stress, and time demands of repeated EUA procedures, while also gaining the reassurance of not passing the familial variant on to future offspring. This study therefore highlights the importance of sustained research and genetic analyses of understudied populations to enhance both knowledge and patient care, globally.

## Supplementary Information

Supplementary Material 1

Supplementary Material 2

## Data Availability

The datasets generated and/or analysed during the current study are available on ClinVar (https:/www.ncbi.nlm.nih.gov/clinvar/) under the accession numbers SCV007449574-SCV007449665.

## Abbreviations

Rb: Retinoblastoma
RB1: Retinoblastoma 1
NGS: Next-generation sequencing
SNV: Single nucleotide variant
Indels: Insertions and deletions
CNV: Copy number variant
MS-MLPA: Methylation-specific multiplex ligation-dependent probe amplification
pRb: Retinoblastoma associated protein
WHO: World Health Organization
SA: South Africa
LMICs: Low- and middle-income countries
EUA: Examination under anesthetic
UCT: University of Cape Town
HREC: Human Research Ethics Committee
ISP: Ion sphere particle
BAM: Binary Alignment/Map
VCF: Variant call file
gnomAD: Genome Aggregation Database
IRD: Inherited retinal disease
LOVD: Leiden Open Variation Database
IGV: Integrative Genomics Viewer
ESE: Exonic splicing enhancer
ACMG: American College of Medical Genetics and Genomics
NCBI: National Center for Biotechnology Information
PCR: Polymerase chain reaction
ClinGen: Clinical Genome Resource
MgCl_2_: Magnesium chloride
USD: United States Dollar
ZAR: South African Rand
ExAC: Exome Aggregation Consortium
DIV: Deep intronic variant

## References

[1] Schaiquevich P, Francis JH, Cancela MB, Carcaboso AM, Chantada GL, Abramson DH. Treatment of retinoblastoma: what is the latest and what is the future. Front Oncol. 2022;12:822330. 10.3389/fonc.2022.822330. 35433448 10.3389/fonc.2022.822330 PMC9010858

[2] Lohmann DR. RB1 gene mutations in retinoblastoma. Hum Mutat. 1999;14:283–810.1002/(SICI)1098-1004(199910)14:4%3C;283::AID-HUMU2%3E;3.0.CO;2-J. 10502774 10.1002/(SICI)1098-1004(199910)14:4<283::AID-HUMU2>3.0.CO;2-J

[3] Kivelä T. The epidemiological challenge of the most frequent eye cancer: retinoblastoma, an issue of birth and death. Br J Ophthalmol. 2009;93:1129–31. 10.1136/bjo.2008.150292. 19704035 10.1136/bjo.2008.150292

[4] Stuart KV, Shepherd DJ, Kruger M, Singh E. The incidence of retinoblastoma in South Africa: findings from the South African National Cancer Registry (2004–2018). Ophthalmic Epidemiol. 2021;29:681–7. 10.1080/09286586.2021.2013900. 34935580 10.1080/09286586.2021.2013900

[5] Ancona-Lezama D, Dalvin LA, Shields CL. Modern treatment of retinoblastoma: a 2020 review. Indian J Ophthalmol. 2020;68:2356–65. 10.4103/ijo.IJO_721_20. 33120616 10.4103/ijo.IJO_721_20 PMC7774148

[6] The Global Retinoblastoma Group. The global retinoblastoma outcome study: a prospective, cluster-based analysis of 4064 patients from 149 countries. Lancet Glob Heal. 2022;10:e1128–40. 10.1016/S2214-109X(22)00250-9. 10.1016/S2214-109X(22)00250-9 PMC9397647 35839812

[7] Kaewkhaw R, Rojanaporn D. Retinoblastoma: etiology, modeling, and treatment. Cancers (Basel). 2020;12:2304. 10.3390/cancers12082304. 32824373 10.3390/cancers12082304 PMC7465685

[8] Knudson AG. Mutation and cancer: statistical study of retinoblastoma. Proc Natl Acad Sci U S A. 1971;68:820–3. 10.1073/pnas.68.4.820. 5279523 10.1073/pnas.68.4.820 PMC389051

[9] Friend SH, Bernards R, Rogelj S, Weinberg RA, Rapaport JM, Albert DM, et al. A human DNA segment with properties of the gene that predisposes to retinoblastoma and osteosarcoma. Nature. 1986;323:643–6. 10.1038/323643a0. 2877398 10.1038/323643a0

[10] Yao Y, Gu X, Xu X, Ge S, Jia R. Novel insights into RB1 mutation. Cancer Lett. 2022;547:215870. 10.1016/j.canlet.2022.215870. 35964818 10.1016/j.canlet.2022.215870

[11] Fokkema IFAC, Kroon M, López Hernández JA, Asscheman D, Lugtenburg I, Hoogenboom J, et al. The LOVD3 platform: efficient genome-wide sharing of genetic variants. Eur J Hum Genet. 2021;29:1796–803. 10.1038/s41431-021-00959-x. 34521998 10.1038/s41431-021-00959-x PMC8632977

[12] Lan X, Xu W, Tang X, Ye H, Song X, Lin L, et al. Spectrum of RB1 germline mutations and clinical features in unrelated Chinese patients with retinoblastoma. Front Genet. 2020;11:142. 10.3389/fgene.2020.00142. 32218800 10.3389/fgene.2020.00142 PMC7080181

[13] Parma D, Ferrer M, Luce L, Giliberto F, Szijan I. RB1 gene mutations in Argentine retinoblastoma patients. Implications for genetic counseling. PLoS ONE. 2017;12:e0189736. 10.1371/journal.pone.0189736. 29261756 10.1371/journal.pone.0189736 PMC5738096

[14] Dunn JM, Phillips RA, Zhu X, Becker A, Gallie BL. Mutations in the RB1 gene and their effects on transcription. Mol Cell Biol. 1989;9:4596–604. 10.1128/mcb.9.11.4596-4604.1989. 2601691 10.1128/mcb.9.11.4596-4604.1989 PMC363605

[15] Taylor M, Dehainault C, Desjardins L, Doz F, Levy C, Sastre X, et al. Genotype–phenotype correlations in hereditary familial retinoblastoma. Hum Mutat. 2007;28:284–93. 10.1002/humu.20443. 17096365 10.1002/humu.20443

[16] Freeman N, Meyer D. Towards early detection of retinoblastoma. South Afr Med J. 2014;104:8741. 10.7196/SAMJ.8741. 10.7196/SAMJ.8741 28375074

[17] Fletcher O, Easton D, Anderson K, Gilham C, Jay M, Peto J. Lifetime risks of common cancers among retinoblastoma survivors. J Natl Cancer Inst. 2004;96:357–63. 10.1093/jnci/djh058. 14996857 10.1093/jnci/djh058

[18] Devarajan B, Prakash L, Kannan TR, Abraham AA, Kim U, Muthukkaruppan V, et al. Targeted next generation sequencing of RB1 gene for the molecular diagnosis of retinoblastoma. BMC Cancer. 2015;15:320. 10.1186/s12885-015-1340-8. 25928201 10.1186/s12885-015-1340-8 PMC4415345

[19] Ahani A, Akbari MT, Saliminejad K, Behnam B, Akhondi MM, Vosoogh P, et al. Screening for large rearrangements of the RB1 gene in Iranian patients with retinoblastoma using multiplex ligation-dependent probe amplification. Mol Vis. 2013;19:454–62. 23441118 PMC3580967

[20] Ahani A, Behnam B, Khorshid K, H.R., and, Akbari MT. RB1 gene mutations in Iranian patients with retinoblastoma: report of four novel mutations. Cancer Genet. 2011;204:316–22. 10.1016/j.cancergen.2011.04.007. 21763628 10.1016/j.cancergen.2011.04.007

[21] Grotta S, D’Elia G, Scavelli R, Genovese S, Surace C, Sirleto P, et al. Advantages of a next generation sequencing targeted approach for the molecular diagnosis of retinoblastoma. BMC Cancer. 2015;15:841. 10.1186/s12885-015-1854-0. 26530098 10.1186/s12885-015-1854-0 PMC4632486

[22] Goolam S, Kana H, Welsh N, Wainwright L, Poole J, Mayet I. A 20-year retrospective review of retinoblastoma at two tertiary academic hospitals in Johannesburg, South Africa. Ocul Oncol Pathol. 2018;4:170–5. 10.1159/000481508. 29765949 10.1159/000481508 PMC5939678

[23] Kruger M, Reynders D, Omar F, Schoeman J, Wedi O, Harvey J. Retinoblastoma outcome at a single institution in South Africa. South Afr Med J. 2014;104:859–63. 10.7196/samj.8255. 10.7196/samj.8255 26042269

[24] Park S, Essuman YA, Sherief ST, Soliman SE, Essuman VA, Dimaras H. Retinoblastoma research in Africa: a scoping review. Pediatr Blood Cancer. 2025;72:e32074. 10.1002/pbc.32074. 41017280 10.1002/pbc.32074

[25] World Medical Association. World Medical Association Declaration of Helsinki, ethical principles for medical research involving human subjects. JAMA. 2013;310:2191–4. 10.1001/jama.2013.281053. 24141714 10.1001/jama.2013.281053

[26] Miller SA, Dykes DD, Polesky HF. A simple salting out procedure for extracting DNA from human nucleated cells. Nucleic Acids Res. 1988;16:1215. 10.1093/nar/16.3.1215. 3344216 10.1093/nar/16.3.1215 PMC334765

[27] Midgley N, Rebello G, Holtes LK, Ramesar R, Roberts L. Screening of inherited retinal disease patients in a low-resource setting using an augmented next-generation sequencing panel. Mol Genet Genomic Med. 2024;12:e70046. 10.1002/mgg3.70046. 39676705 10.1002/mgg3.70046 PMC11647334

[28] Landrum MJ, Lee JM, Benson M, Brown GR, Chao C, Chitipiralla S, et al. ClinVar: improving access to variant interpretations and supporting evidence. Nucleic Acids Res 46. 2018;D1062–7. 10.1093/nar/gkx1153. 10.1093/nar/gkx1153 PMC5753237 29165669

[29] Robinson JT, Thorvaldsdottir H, Turner D, Mesirov JP. igv.js: an embeddable JavaScript implementation of the Integrative Genomics Viewer (IGV). Bioinformatics. 2023;39(btac830). 10.1002/mgg3.70046. 10.1093/bioinformatics/btac830 PMC9825295 36562559

[30] Ng PC, Henikoff S. Predicting deleterious amino acid substitutions. Genome Res. 2001;11:863–74. 10.1101/gr.176601. 11337480 10.1101/gr.176601 PMC311071

[31] Adzhubei IA, Schmidt S, Peshkin L, Ramensky VE, Gerasimova A, Bork P, et al. A method and server for predicting damaging missense mutations. Nat Methods. 2010;7:248–9. 10.1038/nmeth0410-248. 20354512 10.1038/nmeth0410-248 PMC2855889

[32] Grantham R. Amino acid difference formula to help explain protein evolution. Sci (80-). 1974;185:862–4. 10.1126/science.185.4154.862. 10.1126/science.185.4154.862 4843792

[33] Rogers MF, Shihab HA, Mort M, Cooper DN, Gaunt TR, Campbell C. FATHMM-XF: accurate prediction of pathogenic point mutations via extended features. Bioinformatics. 2018;34:511–3. 10.1093/bioinformatics/btx536. 28968714 10.1093/bioinformatics/btx536 PMC5860356

[34] Pollard KS, Hubisz MJ, Rosenbloom KR, Siepel A. Detection of nonneutral substitution rates on mammalian phylogenies. Genome Res. 2010;20:110–21. 10.1101/gr.097857.109. 19858363 10.1101/gr.097857.109 PMC2798823

[35] Jaganathan K, Kyriazopoulou Panagiotopoulou S, McRae JF, Darbandi SF, Knowles D, Li YI, et al. Predicting splicing from primary sequence with deep learning. Cell. 2019;176:535–e54824. 10.1016/j.cell.2018.12.015. 30661751 10.1016/j.cell.2018.12.015

[36] Lefter M, Vis JK, Vermaat M, den Dunnen JT, Taschner PEM, Laros JFJ. Mutalyzer 2: next generation HGVS nomenclature checker. Bioinformatics. 2021;37:2811–7. 10.1093/bioinformatics/btab051. 33538839 10.1093/bioinformatics/btab051 PMC8479679

[37] Richards S, Aziz N, Bale S, Bick D, Das S, Gastier-Foster J. Standards and guidelines for the interpretation of sequence variants: a joint consensus recommendation of the American College of Medical Genetics and Genomics and the Association for Molecular Pathology. Genet Med. 2015;17:405–24. 10.1038/gim.2015.30. 25741868 10.1038/gim.2015.30 PMC4544753

[38] Dyer SC, Austine-Orimoloye O, Azov AG, Barba M, Barnes I, Barrera-Enriquez VP, et al. Ensembl 2025. Nucleic Acids Res 53. 2025;D948–57. 10.1093/nar/gkae1071. 10.1093/nar/gkae1071 PMC11701638 39656687

[39] Untergasser A, Cutcutache I, Koressaar T, Ye J, Faircloth BC, Remm M, et al. Primer3–new capabilities and interfaces. Nucleic Acids Res. 2012;40:e115. 10.1093/nar/gks596. 22730293 10.1093/nar/gks596 PMC3424584

[40] Koressaar T, Remm M. Enhancements and modifications of primer design program Primer3. Bioinformatics. 2007;23:1289–91. 10.1093/bioinformatics/btm091. 17379693 10.1093/bioinformatics/btm091

[41] Ye J, Coulouris G, Zaretskaya I, Cutcutache I, Rozen S, Madden TL. Primer-BLAST: a tool to design target-specific primers for polymerase chain reaction. BMC Bioinformatics. 2012;13:134. 10.1186/1471-2105-13-134. 22708584 10.1186/1471-2105-13-134 PMC3412702

[42] Perez G, Raney BJ, Barber GP, Benet-Pagès A, Casper J, Clawson H, Diekhans M, et al. The UCSC Genome Browser database: 2025 update. Nucleic Acids Res 53. 2025;D1243–9. 10.1093/nar/gkae974. 10.1093/nar/gkae974 PMC11701590 39460617

[43] Rensburg R. (2021). Healthcare in South Africa: how inequity is contributing to inefficiency. Conversat. Africa. https://www.wits.ac.za/news/latest-news/opinion/2021/2021-07/healthcare-in-south-africa-how-inequity-is-contributing-to-inefficiency.html

[44] Cowell JK, Smith T, Bia B. Frequent constitutional C to T mutations in CGA-arginine codons in the RB1 gene produce premature stop codons in patients with bilateral (hereditary) retinoblastoma. Eur J Hum Genet. 1994;2:281–90. 10.1159/000472372. 7704558 10.1159/000472372

[45] Zhang K, Nowak I, Rushlow D, Gallie BL, Lohmann DR. Patterns of missplicing caused by RB1 gene mutations in patients with retinoblastoma and association with phenotypic expression. Hum Mutat. 2008;29:475–84. 10.1002/humu.20664. 18181215 10.1002/humu.20664

[46] Pejaver V, Byrne AB, Feng BJ, Pagel KA, Mooney SD, Karchin R, O’Donnell-Luria A, et al. Calibration of computational tools for missense variant pathogenicity classification and ClinGen recommendations for PP3/BP4 criteria. Am J Hum Genet. 2022;109:2163–77. 10.1016/j.ajhg.2022.10.013. 36413997 10.1016/j.ajhg.2022.10.013 PMC9748256

[47] Henke W, Herdel K, Jung K, Schnorr D, Loening SA. Betaine improves the PCR amplification of GC-rich DNA sequences. Nucleic Acids Res. 1997;25:3957–8. 10.1093/nar/25.19.3957. 9380524 10.1093/nar/25.19.3957 PMC146979

[48] Wong KHY, Ma W, Wei CY, Yeh EC, Lin WJ, Wang EHF, et al. Towards a reference genome that captures global genetic diversity. Nat Commun. 2020;11:5482. 10.1038/s41467-020-19311-w. 33127893 10.1038/s41467-020-19311-w PMC7599213

[49] Kugalingam N, De Silva D, Abeysekera H, Nanayakkara S, Tirimanne S, Ranaweera D, et al. RB1 screening of retinoblastoma patients in Sri Lanka using targeted next generation sequencing (NGS) and gene ratio analysis copy enumeration PCR (GRACE-PCR). BMC Med Genomics. 2023;16:279. 10.1186/s12920-023-01721-6. 37932687 10.1186/s12920-023-01721-6 PMC10626775

[50] Gomez-Mariano G, Hernandez-SanMiguel E, Fernandez-Prieto M, Ramos del Saz S, Baladrón B, Mielu LM, et al. Mosaicism and intronic variants in RB1 gene revealed by next generation sequencing in a cohort of Spanish retinoblastoma patients. Exp Eye Res 251. 2025. 10.1016/j.exer.2025.110233. 10.1016/j.exer.2025.110233 39778672

[51] Rojanaporn D, Chitphuk S, Iemwimangsa N, Chareonsirisuthigul T, Saengwimol D, Aroonroch R, et al. Germline RB1 mutation in retinoblastoma patients: detection methods and implication in tumor focality. Transl Vis Sci Technol. 2022;11:30. 10.1167/tvst.11.9.30. 36173648 10.1167/tvst.11.9.30 PMC9527333

[52] Tomar S, Sethi R, Sundar G, Quah TC, Quah BL, Lai PS. Mutation spectrum of RB1 mutations in retinoblastoma cases from Singapore with implications for genetic management and counselling. PLoS ONE. 2017;12:e0178776. 10.1371/journal.pone.0178776. 28575107 10.1371/journal.pone.0178776 PMC5456385

[53] Kiran VS, Kannabiran C, Chakravarthi K, Vemuganti GK, Honavar SG. Mutational screening of the RB1 gene in Indian patients with retinoblastoma reveals eight novel and several recurrent mutations. Hum Mutat. 2003;22:339. 10.1002/humu.9181. 12955724 10.1002/humu.9181

[54] Price EA, Price K, Kolkiewicz K, Hack S, Reddy MA, Hungerford JL, et al. Spectrum of RB1 mutations identified in 403 retinoblastoma patients. J Med Genet. 2014;51:208–14. 10.1136/jmedgenet-2013-101821. 24225018 10.1136/jmedgenet-2013-101821

[55] Sagi M, Frenkel A, Eilat A, Weinberg N, Frenkel S, Pe’er J, et al. Genetic screening in patients with retinoblastoma in Israel. Fam Cancer. 2015;14:471–80. 10.1007/s10689-015-9794-z. 25754945 10.1007/s10689-015-9794-z

[56] Dommering CJ, Mol BM, Moll AC, Burton M, Cloos J, Dorsman JC, et al. RB1 mutation spectrum in a comprehensive nationwide cohort of retinoblastoma patients. J Med Genet. 2014;51:366–74. 10.1136/jmedgenet-2014-102264. 24688104 10.1136/jmedgenet-2014-102264

[57] Chow KN, Dean DC. Domains A and B in the Rb pocket interact to form a transcriptional repressor motif. Mol Cell Biol. 1996;16:4862–8. 10.1128/mcb.16.9.4862. 8756645 10.1128/mcb.16.9.4862 PMC231488

[58] Gupta H, Malaichamy S, Mallipatna A, Murugan S, Jeyabalan N, Babu S, et al. Retinoblastoma genetics screening and clinical management. BMC Med Genomics. 2021;14:188. 10.1186/s12920-021-01034-6. 34294096 10.1186/s12920-021-01034-6 PMC8296631

[59] Singh J, Mishra A, Pandian AJ, Mallipatna AC, Khetan V, Sripriya S, et al. Next-generation sequencing-based method shows increased mutation detection sensitivity in an Indian retinoblastoma cohort. Mol Vis. 2016;16:1036–47. PMC4985049 27582626

[60] Mallipatna A, Marino M, Singh AD. Genetics of retinoblastoma. Asia-Pacific J Ophthalmol. 2016;5:260–4. 10.1097/APO.0000000000000219. 10.1097/APO.0000000000000219 27488068

[61] Dryja TP, Rapaport J, McGee TL, Nork TM, Schwartz TL. Molecular etiology of low-penetrance retinoblastoma in two pedigrees. Am J Hum Genet. 1993;52:1122–8. 8099255 PMC1682279

[62] Kontic M, Palacios I, Gámez Á, Camino I, Latkovic Z, Rasic D, et al. New RB1 oncogenic mutations and intronic polymorphisms in Serbian retinoblastoma patients: Genetic counseling implications. J Hum Genet. 2006;51:909–13. 10.1007/s10038-006-0036-y. 16972022 10.1007/s10038-006-0036-y

[63] Le Gall J, Dehainault C, Benoist C, Matet A, Lumbroso-Le Rouic L, Aerts I, Jiménez I, et al. Highly sensitive detection method of retinoblastoma genetic predisposition and biomarkers. J Mol Diagnostics. 2021;23:1714–21. 10.1016/j.jmoldx.2021.08.014. 10.1016/j.jmoldx.2021.08.014 34656762

[64] Mitter D, Ullmann R, Muradyan A, Klein-Hitpaß L, Kanber D, Õunap K, et al. Genotype-phenotype correlations in patients with retinoblastoma and interstitial 13q deletions. Eur J Hum Genet. 2011;19:947–58. 10.1038/ejhg.2011.58. 21505449 10.1038/ejhg.2011.58 PMC3179359

[65] Dehainault C, Garancher A, Castéra L, Cassoux N, Aerts I, Doz F, et al. The survival gene MED4 explains low penetrance retinoblastoma in patients with large RB1 deletion. Hum Mol Genet. 2014;23:5243–50. 10.1093/hmg/ddu245. 24858910 10.1093/hmg/ddu245

[66] Kanber D, Berulava T, Ammerpohl O, Mitter D, Richter J, Siebert R, et al. The human retinoblastoma gene is imprinted. PLoS Genet. 2009;5:e1000790. 10.1371/journal.pgen.1000790. 20041224 10.1371/journal.pgen.1000790 PMC2791201

[67] Eloy P, Dehainault C, Sefta M, Aerts I, Doz F, Cassoux N, et al. A parent-of-origin effect impacts the phenotype in low penetrance retinoblastoma families segregating the c.1981C > T/p.Arg661Trp mutation of RB1. PLoS Genet. 2016;12:e1005888. 10.1371/journal.pgen.1005888. 26925970 10.1371/journal.pgen.1005888 PMC4771840

[68] Chen Z, Moran K, Richards-Yutz J, Toorens E, Gerhart D, Ganguly T, et al. Enhanced sensitivity for detection of low-level germline mosaic RB1 mutations in sporadic retinoblastoma cases using deep semiconductor sequencing. Hum Mutat. 2014;35:384–91. 10.1002/humu.22488. 24282159 10.1002/humu.22488 PMC4112364

[69] Rushlow DE, Mol BM, Kennett JY, Yee S, Pajovic S, Thériault BL, et al. Characterisation of retinoblastomas without RB1 mutations: genomic, gene expression, and clinical studies. Lancet Oncol. 2013;14:327–34. 10.1016/S1470-2045(13)70045-7. 23498719 10.1016/S1470-2045(13)70045-7

[70] Price EA, Patel R, Scheimberg I, Kotiloglu Karaa E, Sagoo MS, Reddy MA, et al. MYCN amplification levels in primary retinoblastoma tumors analyzed by multiple ligation-dependent probe amplification. Ophthalmic Genet. 2021;42:604–11. 10.1080/13816810.2021.1923038. 34003079 10.1080/13816810.2021.1923038

[71] Afshar AR, Pekmezci M, Bloomer MM, Cadenas NJ, Stevers M, Banerjee A, et al. Next-generation sequencing of retinoblastoma identifies pathogenic alterations beyond RB1 inactivation that correlate with aggressive histopathologic features. Ophthalmology. 2020;127:804–13. 10.1016/j.ophtha.2019.12.005. 32139107 10.1016/j.ophtha.2019.12.005 PMC7246167

[72] Kooi IE, Mol BM, Massink MPG, Ameziane N, Meijers-Heijboer H, Dommering CJ, et al. (2016). Somatic genomic alterations in retinoblastoma beyond RB1 are rare and limited to copy number changes. Sci. Rep. *6*. 10.1038/srep25264 10.1038/srep25264 PMC4850475 27126562

[73] Schmidt MJ, Prabakar RK, Pike S, Yellapantula V, Peng CC, Kuhn P, et al. Simultaneous copy number alteration and single-nucleotide variation analysis in matched aqueous humor and tumor samples in children with retinoblastoma. Int J Mol Sci. 2023;24:1–13. 10.3390/ijms24108606. 10.3390/ijms24108606 PMC10218537 37239954

[74] Sippel KC, Fraioli RE, Smith GD, Schalkoff ME, Sutherland J, Gallie BL, et al. Frequency of somatic and germ-line mosaicism in retinoblastoma: implications for genetic counseling. Am J Hum Genet. 1998;62:610–9. 10.1086/301766. 9497263 10.1086/301766 PMC1376960

[75] Greger V, Passarge E, Horsthemke B. Somatic mosaicism in patient with bilateral retinoblastoma. Am J Hum Genet. 1990;46:1187–93. 1971154 PMC1683849

[76] Price EA, Sagoo MS, Reddy MA, Onadim Z. An overview of RB1 transcript alterations detected during retinoblastoma genetic screening. Ophthalmic Genet. 2023;45:235–45. 10.1080/13816810.2023.2270570. 37932244 10.1080/13816810.2023.2270570

[77] Dehainault C, Michaux D, Pagès-Berhouet S, Caux-Moncoutier V, Doz F, et al. A deep intronic mutation in the RB1 gene leads to intronic sequence exonisation. Eur J Hum Genet. 2007;15:473–7. 10.1038/sj.ejhg.5201787. 17299438 10.1038/sj.ejhg.5201787

[78] Soliman SE, Racher H, Lambourne M, Matevski D, MacDonald H, Gallie B. A novel deep intronic low penetrance RB1 variant in a retinoblastoma family. Ophthalmic Genet. 2018;39:288–90. 10.1080/13816810.2017.1393828. 29099630 10.1080/13816810.2017.1393828

