# Supplementary material for: Expanding the RB1 variant landscape of heritable retinoblastoma: unlocking precision oncology potential in Southern Africa
Source: BMC Cancer. 2026 Feb 28;26:341. doi: 10.1186/s12885-026-15789-7 (PMC12983656; doi:10.1186/s12885-026-15789-7)
Supplement: Supplementary file 1 — Supplementary Material 1 [file 12885_2026_15789_MOESM1_ESM.docx]

**Expanding the *RB1* variant landscape of heritable retinoblastoma: unlocking precision oncology potential in Southern Africa**

Indiana van Rensburg^1^, Hamzah Mustak^2^, Gameda Benefeld^1^, Lucy Cunnama^3^, Helga Abrahamse-Pillay^4^, Raj Ramesar^1^, and Lisa Roberts^1^

**Affiliations:**

1 - UCT/MRC Genomic and Precision Medicine Research Unit, Division of Human Genetics, University of Cape Town, Cape Town, South Africa

2 - Division of Ophthalmology, Groote Schuur Hospital and University of Cape Town, Cape Town, South Africa

3 - Health Economics Unit and Division, School of Public Health, Faculty of Health Sciences, University of Cape Town, Cape Town, South Africa

4 - Division of Ophthalmology, Tygerberg Hospital and Stellenbosch University, Cape Town, South Africa

**Correspondence:** Lisa Roberts, UCT/MRC Genomic and Precision Medicine Research Unit, Division of Human Genetics, University of Cape Town, Cape Town, South Africa. [lisa.roberts@uct.ac.za](mailto:lisa.roberts@uct.ac.za)

**Supplementary Figures**


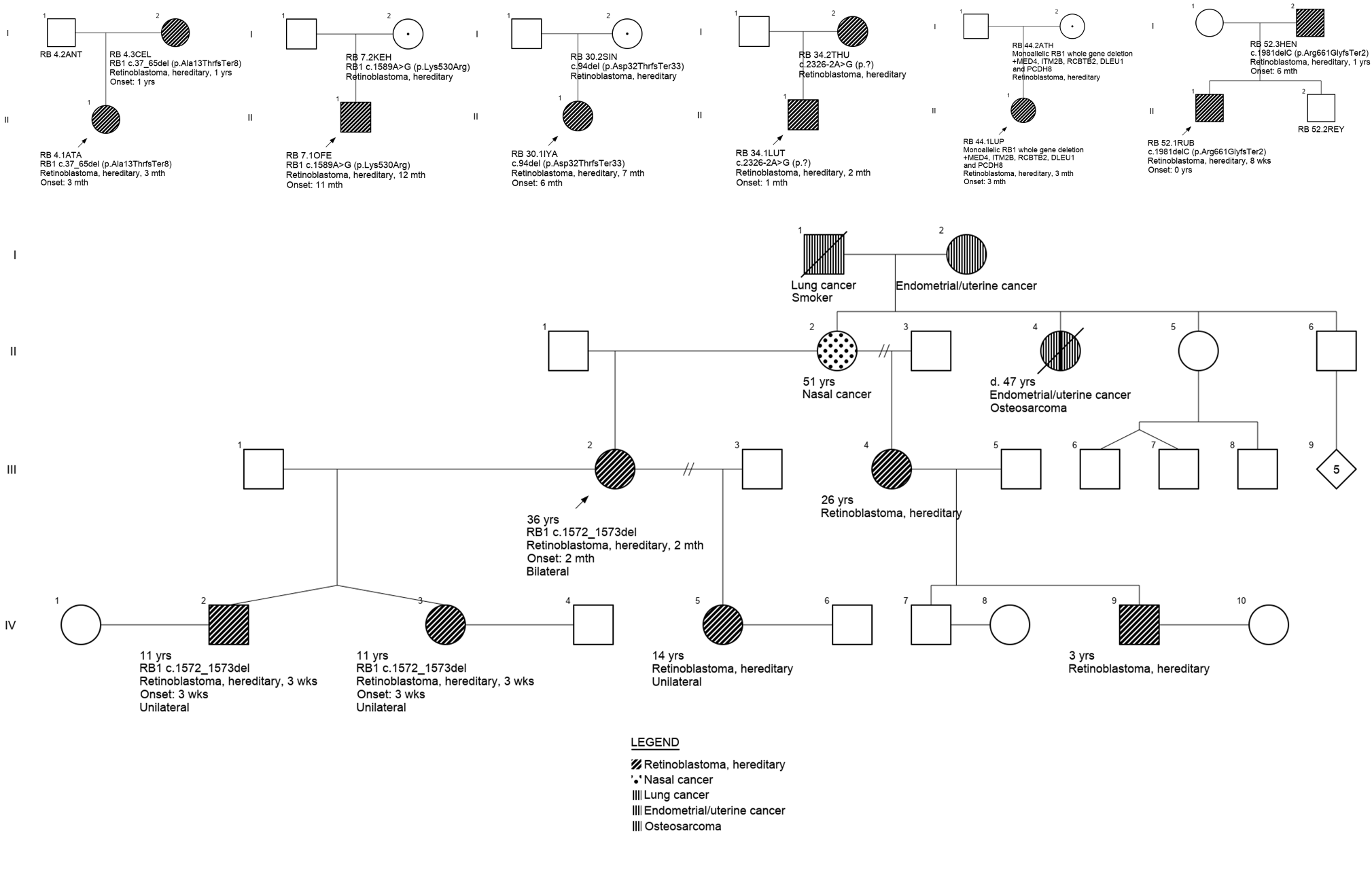


**Figure S1:** **A-G** Shaded symbols represent affected individuals, and unshaded symbols represent healthy individuals. Circles indicate females, squares indicate males, and diamonds represent individuals of unspecified sex. A number within the diamond indicates multiple individuals of unspecified sex. Deceased individuals are marked with a diagonal line, and the proband is indicated by an arrow. Carriers of the familial *RB1* pathogenic variant are represented by a symbol with a dot inside.

**A**

**B**

**C**

**D**

**E**

**F**

**G**


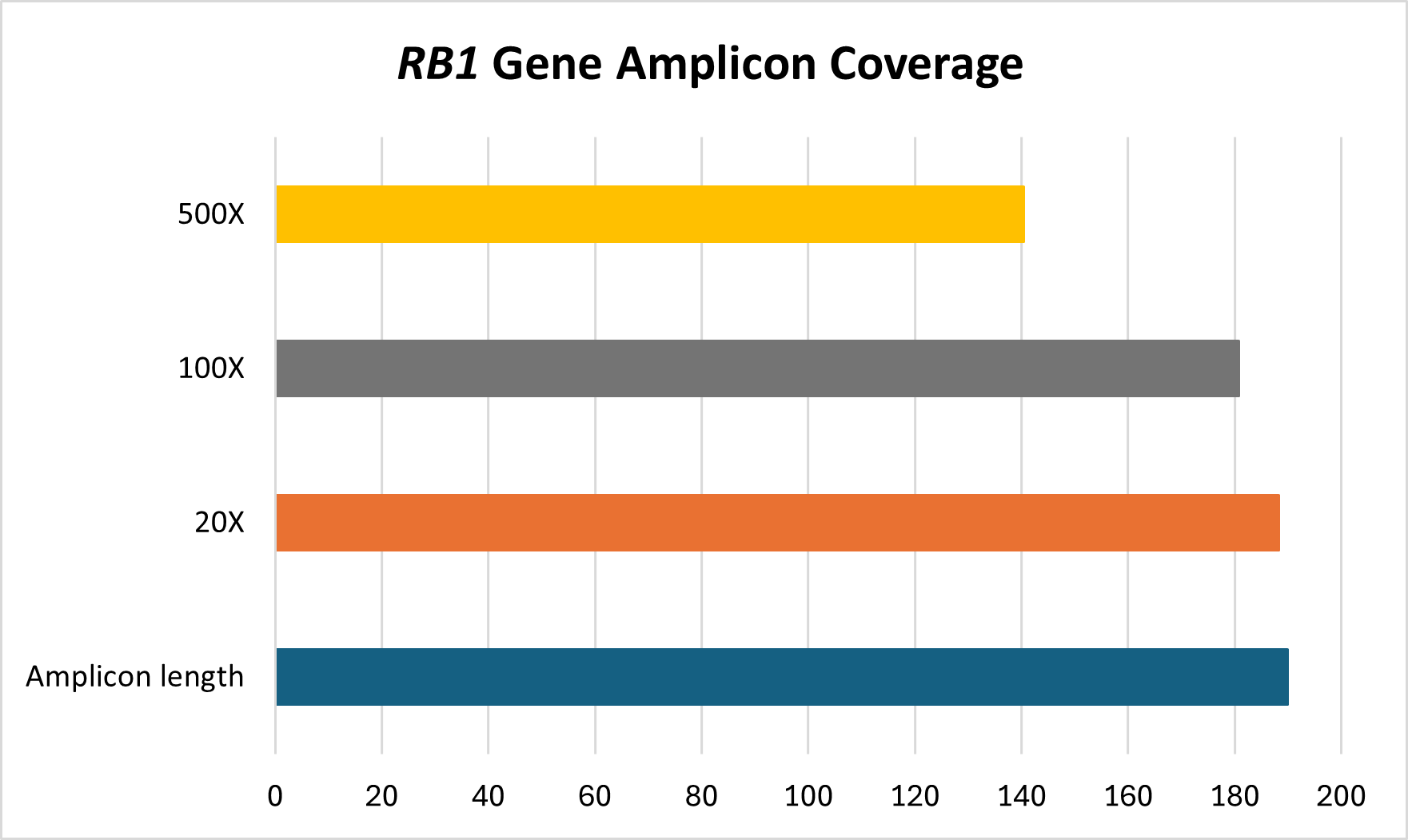


**Figure S2:** *RB1* gene amplicon coverage. 20X, 100X, and 500X: the number of bases of the amplicon target that had at least 20, 100, or 500 reads respectively.


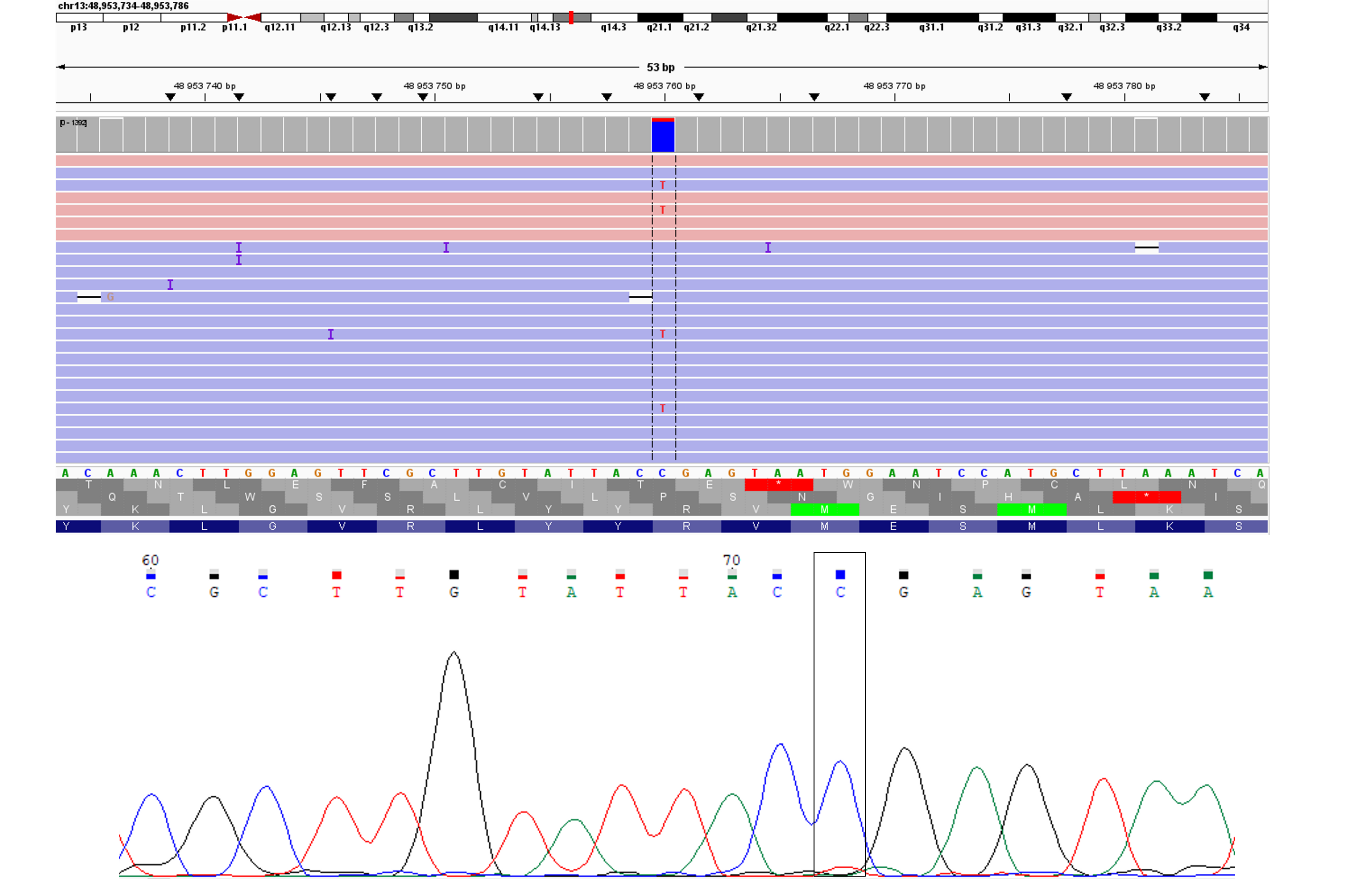


**Figure S3:** Integrative Genomics Viewer (IGV) visualization and Sanger sequencing of the c.1363C>T variant in *RB1* exon 14. **A** IGV visualization illustrates the c.1363C>T variant at an alternative allele fraction of 11% (160/1392 reads). **B** Sanger sequencing did not detect c.1363C>T. The black box indicates the region to which the variant, a single cytosine to thymine change (in exon 14 of *RB1*), is localized (position 72). The forward *RB1* exon 14 primer was used for sequencing and the ABI format chromatographs were analyzed in Chromas.

**A**

**B**

**Figure S4** Sequencing chromatograms illustrating the seven novel frameshift variants identified in this Southern African cohort. **A** c.363dup, **B** c.1515dup, **C** c.771del, **D** c.1572_1573del, **E** c.94del, **F** c.1981del, **G** c.2054_2055insAA.


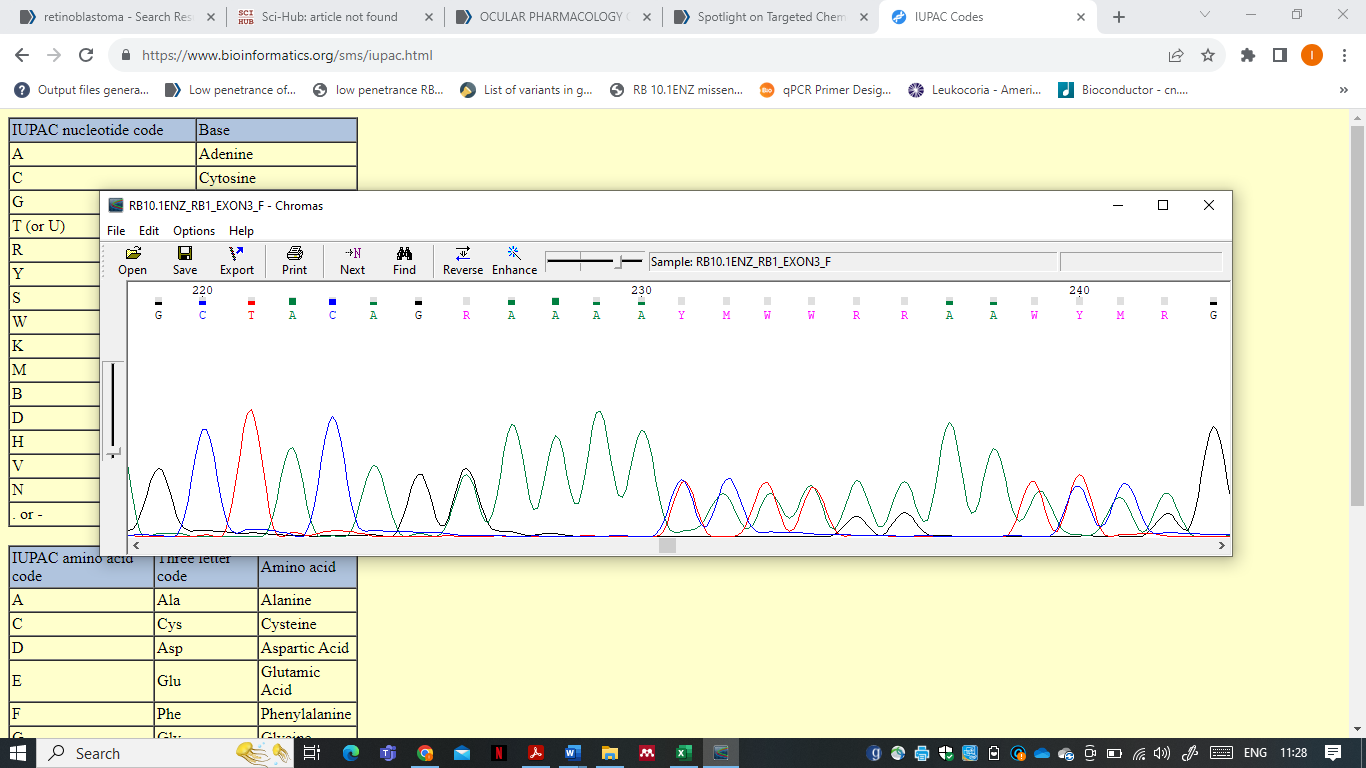


ins G

**A**


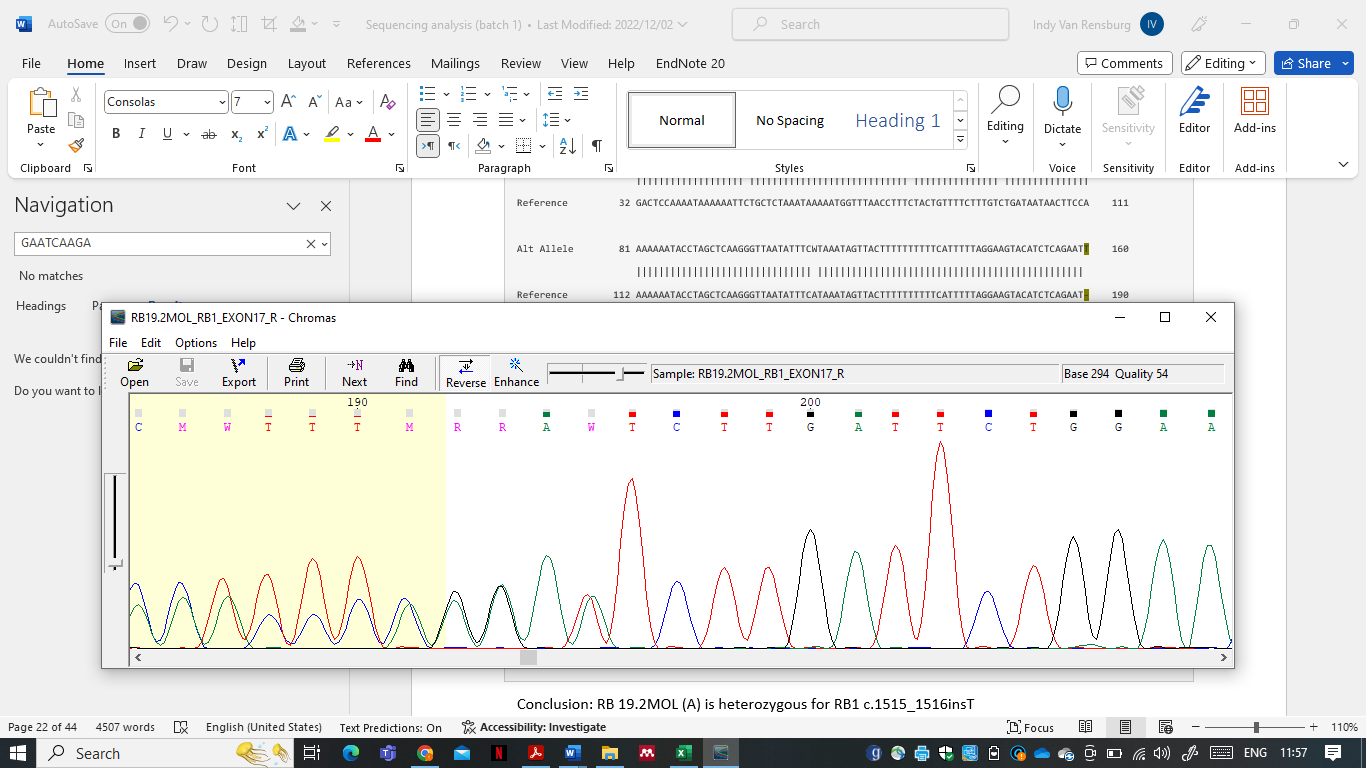


ins T

**B**


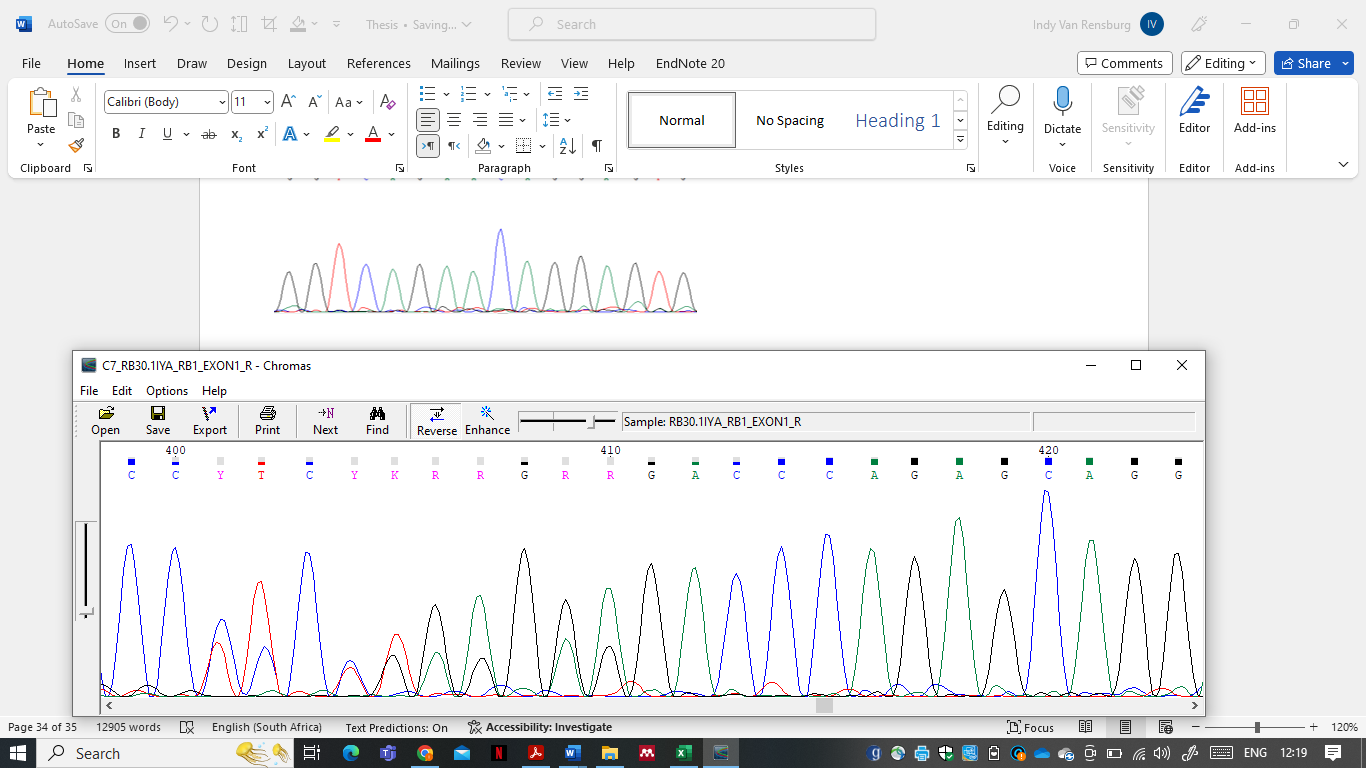


del G

**E**

**D**


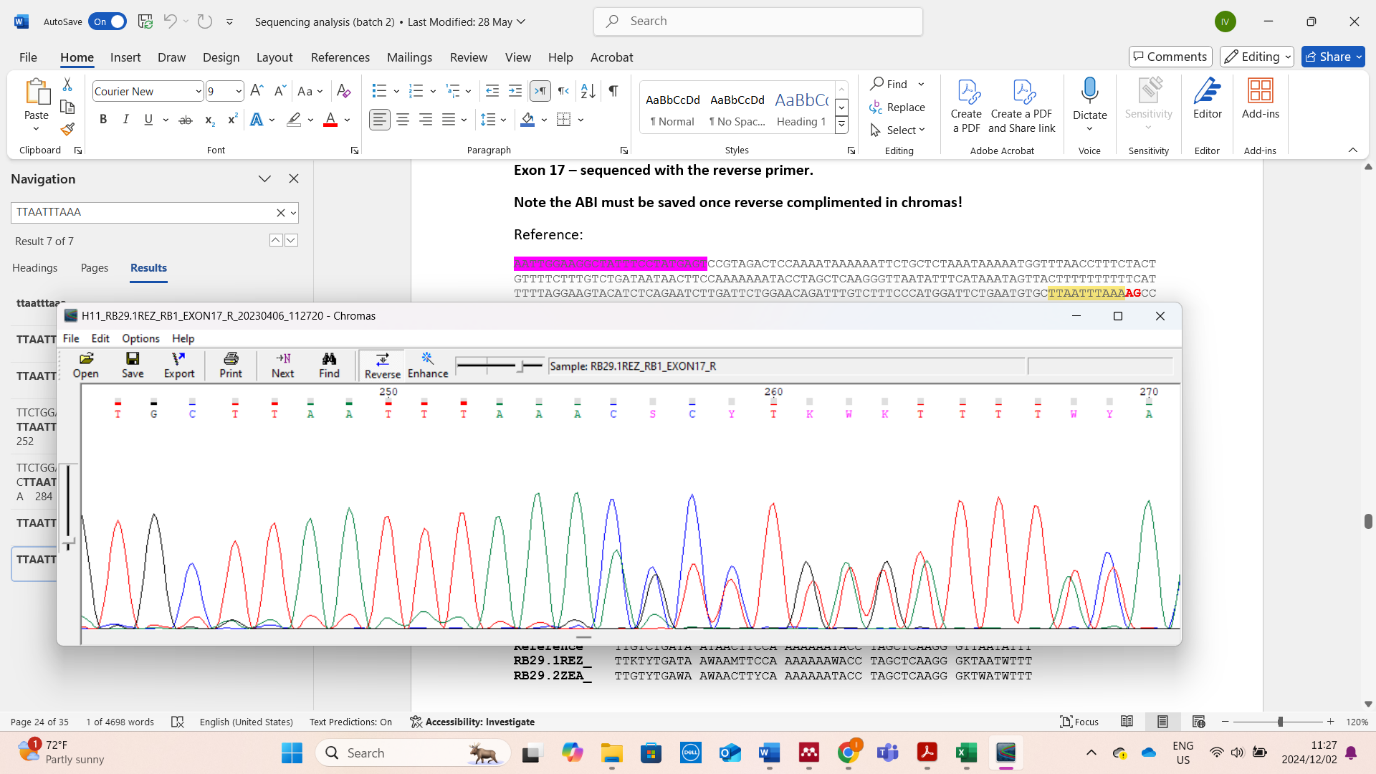


del AG

**F**

del C


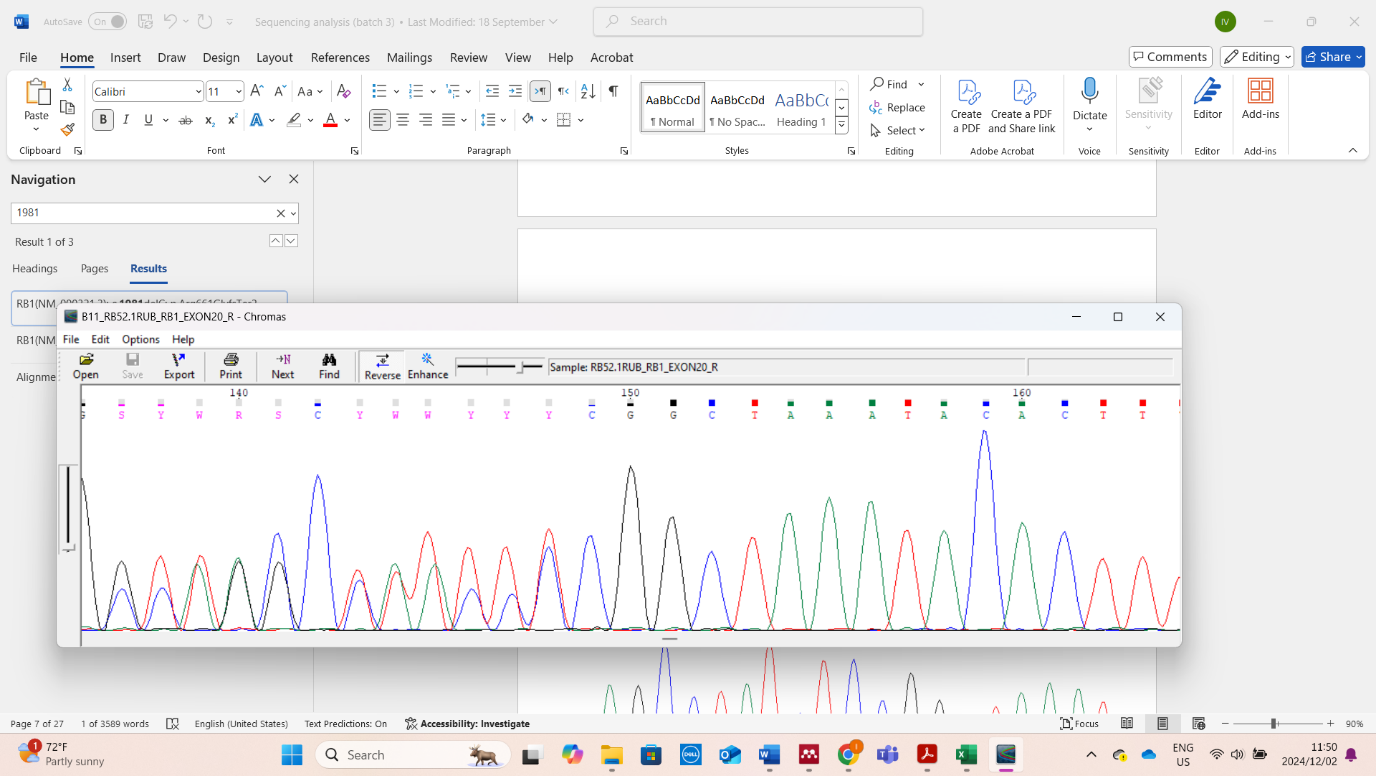

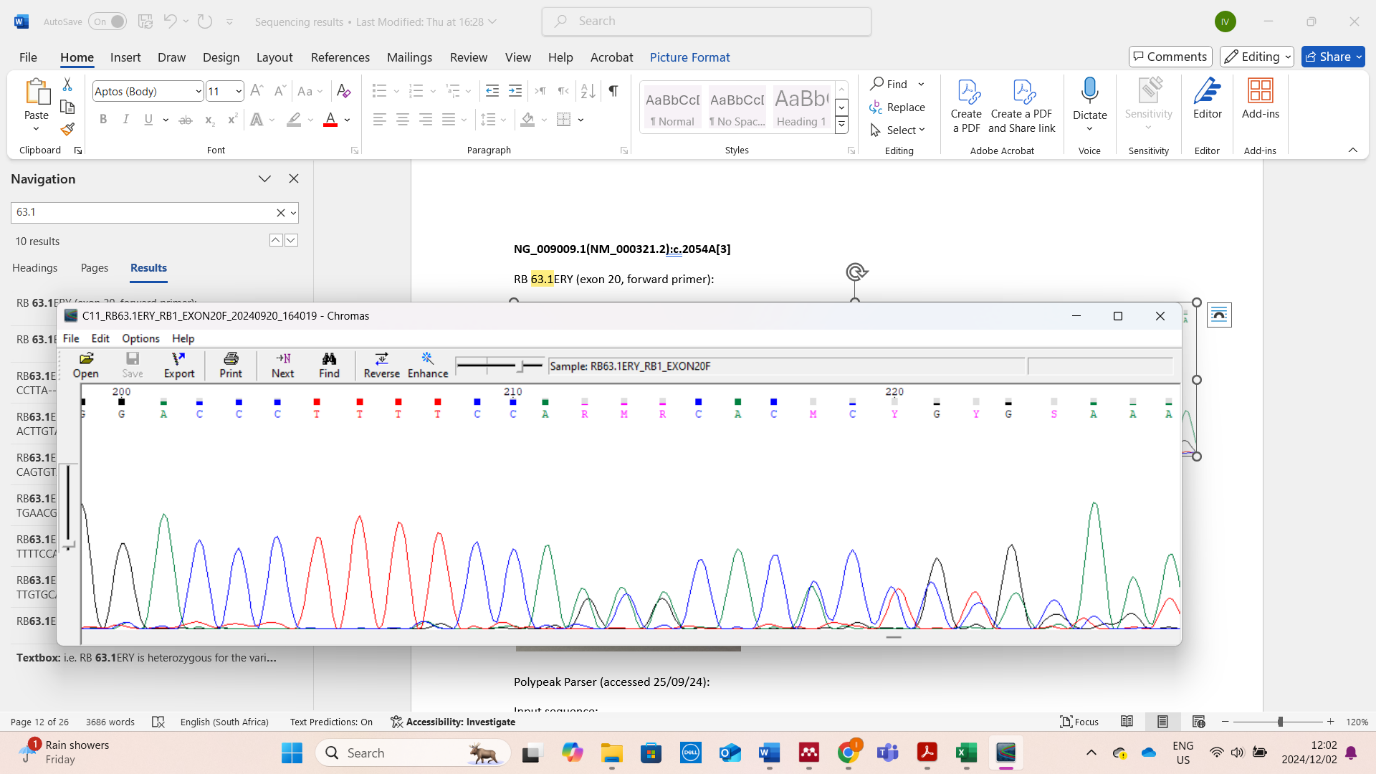


**G**

ins AA

del G

**C**


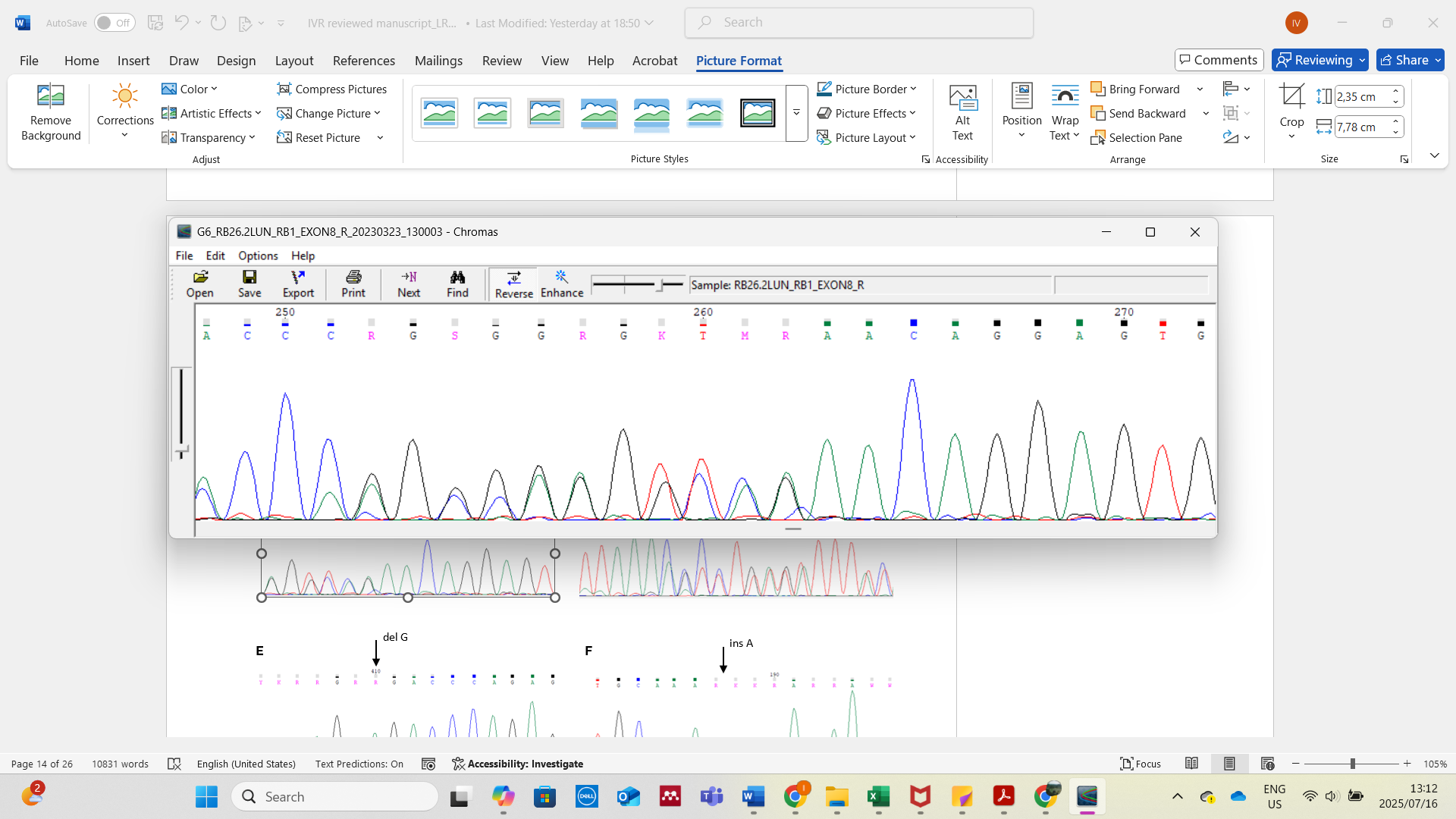


**Figure S5** Integrative Genomics Viewer (IGV) visualization and Sanger sequencing of the c.37_65del variant in *RB1* exon 1. **A** IGV visualization illustrates strand bias, with the deletion detected only in forward strand reads (red). **B** Sequencing with the original exon 1 primers (top panel) shows an apparent homozygous 29-base pair deletion. Using alternative exon 1 primers supplemented with betaine (middle panel) reveals the variant to be heterozygous. Sequencing with the original primers supplemented with betaine (bottom panel) confirms the heterozygous nature of the variant. E1 exon 1.


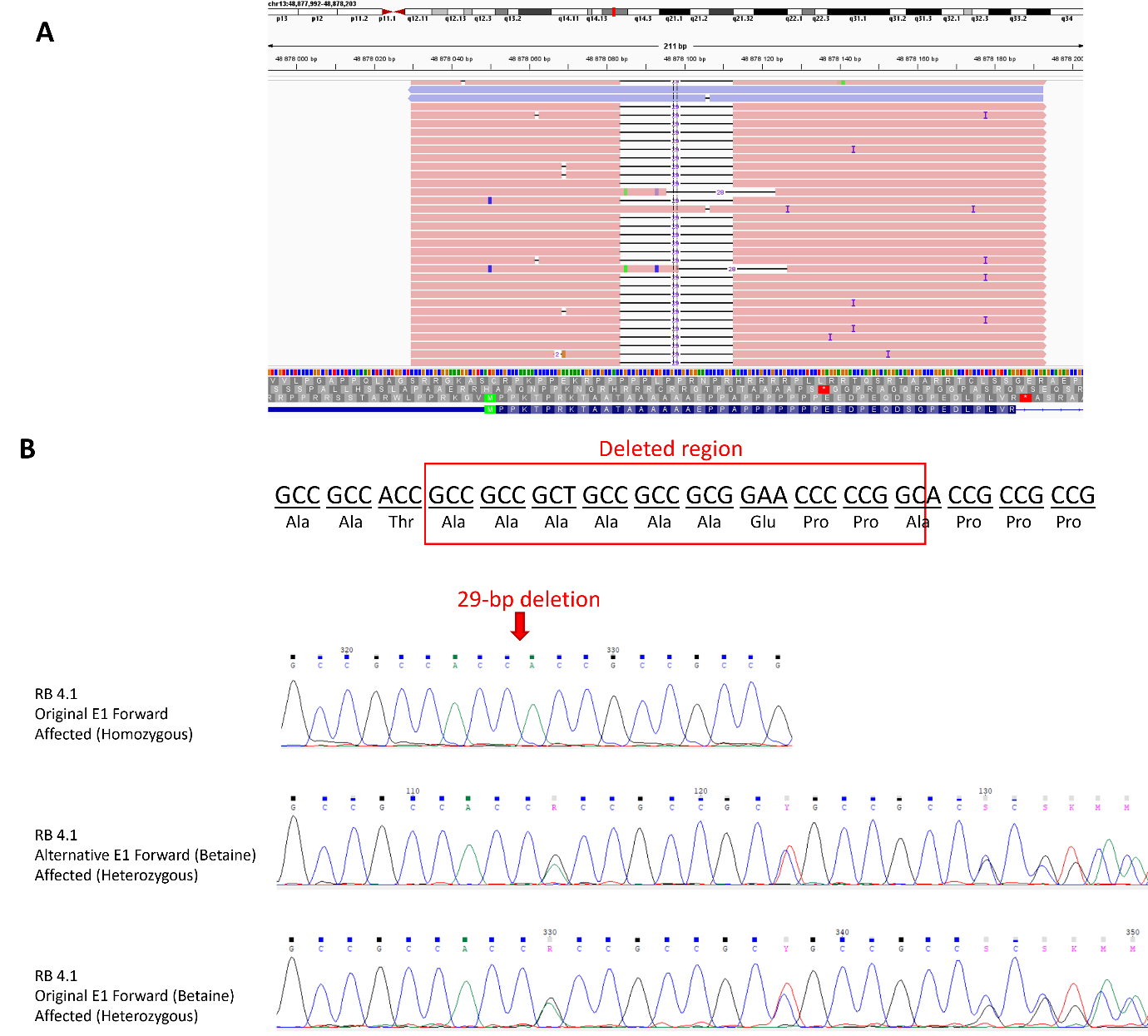


**Figure S6** Representative plots of the methylation-specific multiplex ligation-dependent probe amplification (MS-MLPA) analysis generated using Coffalyser. Each sample is represented by two panels: the upper panel shows the copy number analysis, while the lower panel shows the methylation analysis. Four MS-MLPA probes cover the imprinted locus (CpG85) and provide information on the methylation status of this region (the maternal allele is methylated, and the paternal allele is unmethylated in normal control samples). Hence, the methylation status of CpG85 informs on parental origin of the deletion. Whole gene deletions (including neighboring genes) were detected in patients **A** RB 9.1, **C** RB 24.3, **D** RB 44.1, and **E** RB 50.1. Intragenic deletions of exons 13-14 and exons 24-26 were detected in patients **B** RB 11.1 and **F** RB 60.1, respectively. Patients RB 9.1, RB 12.4, RB 24.3, and RB 50.1 exhibit hypermethylation at CpG85, suggesting paternal inheritance of the deletion. On the other hand, RB 44.1 exhibits hypomethylation at CpG85, consistent with maternal inheritance of the deletion.


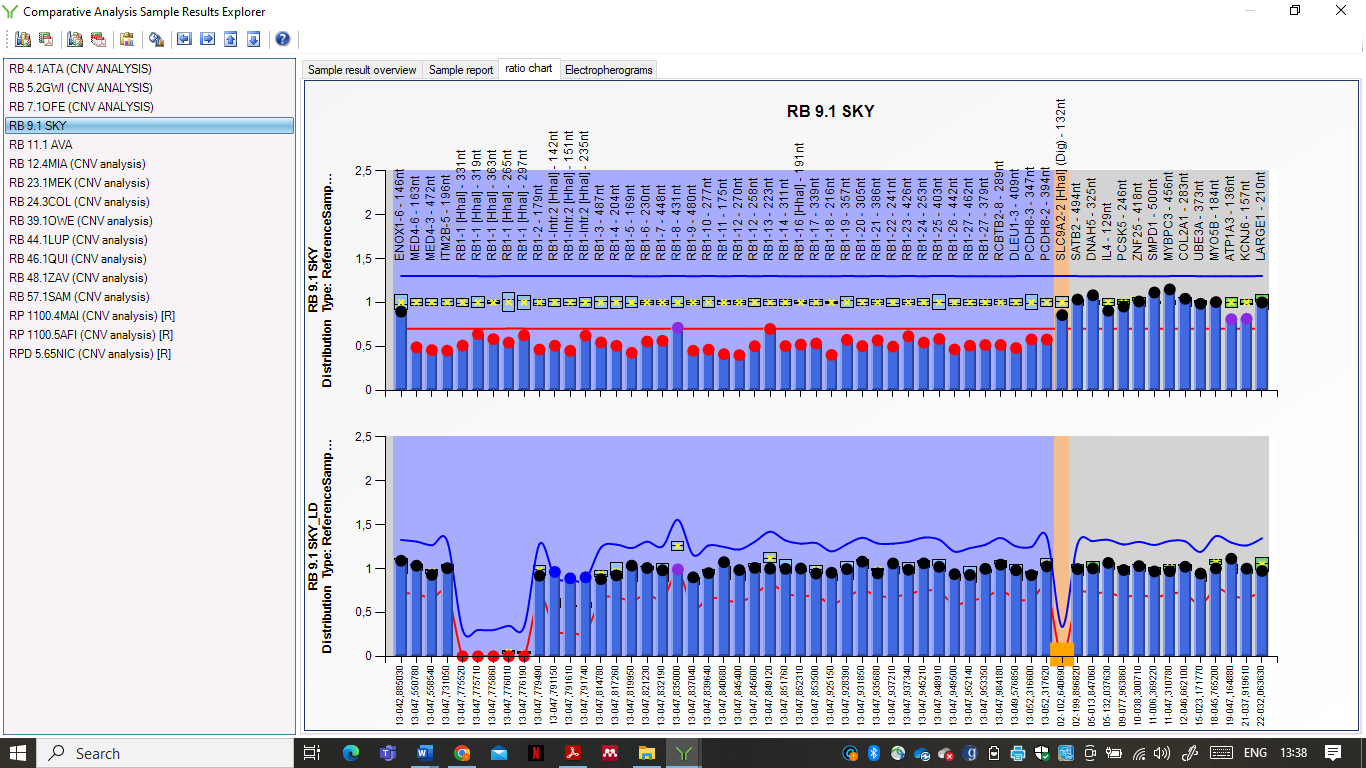


**A**


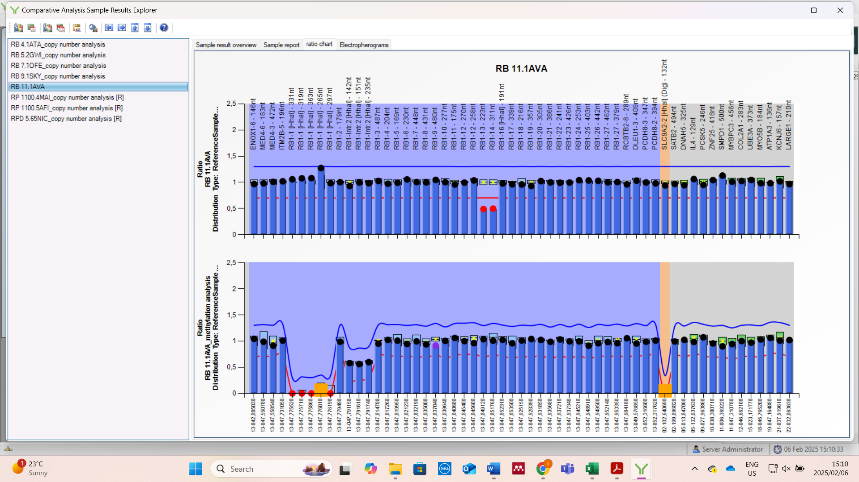


**B**


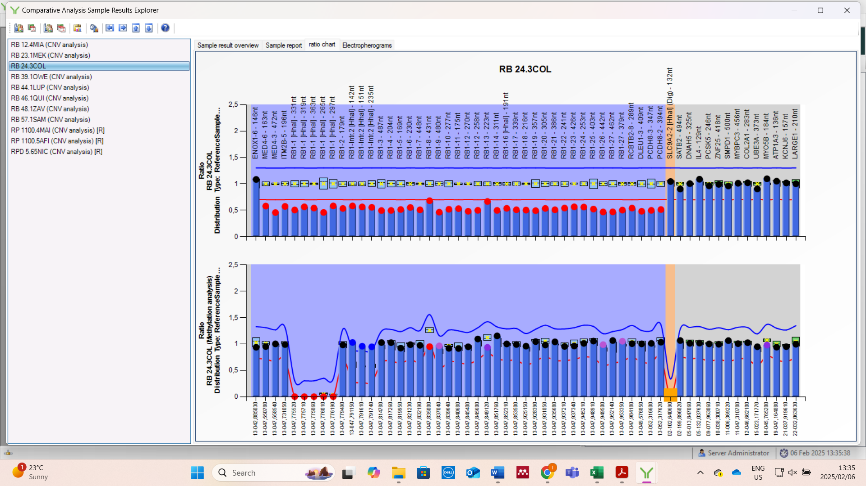


**C**


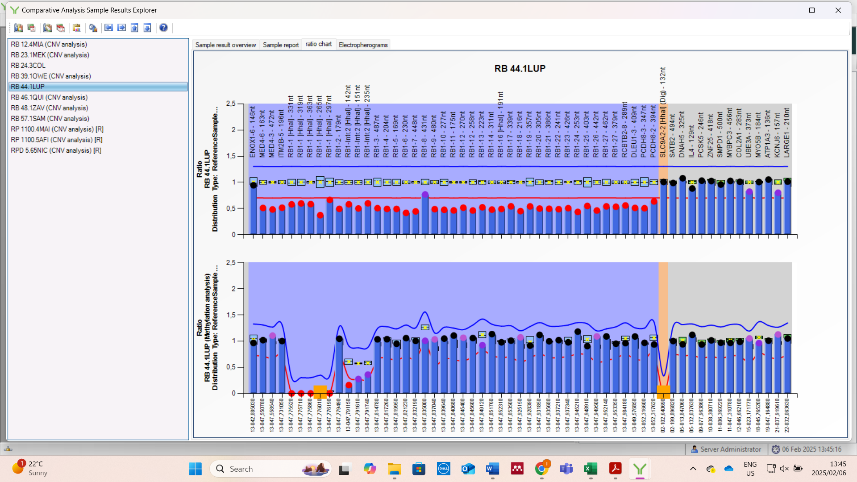


**D**


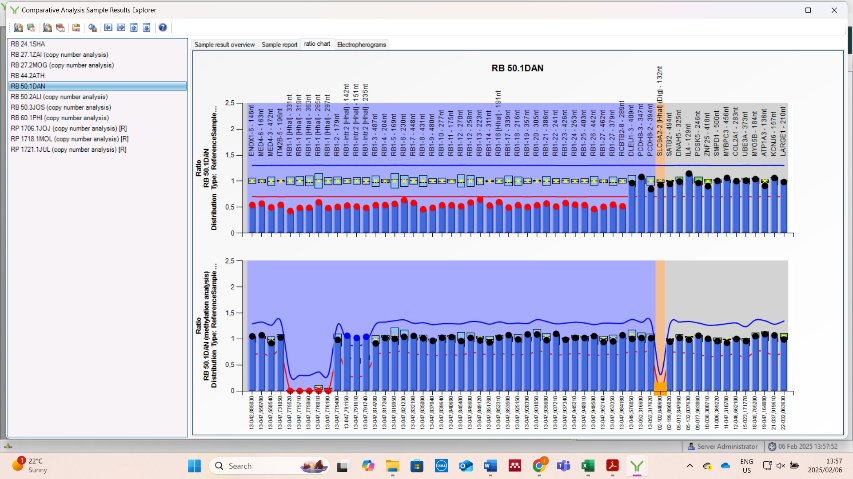


**E**


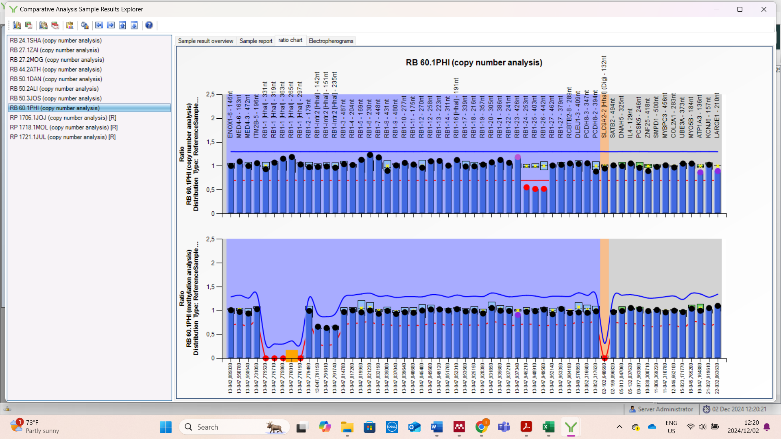


**F**
